# Gaze Behavior in One-Handed Catching and Its Relation with Interceptive Performance: What the Eyes Can't Tell

**DOI:** 10.1371/journal.pone.0119445

**Published:** 2015-03-20

**Authors:** Benedetta Cesqui, Maura Mezzetti, Francesco Lacquaniti, Andrea d'Avella

**Affiliations:** 1 Laboratory of Neuromotor Physiology, IRCCS Santa Lucia Foundation, Rome, Italy; 2 Centre of Space Bio-medicine, University of Rome Tor Vergata, Rome, Italy; 3 Department of Economics and Finance, University of Rome Tor Vergata, Rome, Italy; 4 Department of Systems Medicine, Neuroscience Section, University of Rome, Tor Vergata, Rome, Italy; 5 Department of Biomedical Sciences and Morphological and Functional Images, University of Messina, Messina, Italy; University of Rome, ITALY

## Abstract

In ball sports, it is usually acknowledged that expert athletes track the ball more accurately than novices. However, there is also evidence that keeping the eyes on the ball is not always necessary for interception. Here we aimed at gaining new insights on the extent to which ocular pursuit performance is related to catching performance. To this end, we analyzed eye and head movements of nine subjects catching a ball projected by an actuated launching apparatus. Four different ball flight durations and two different ball arrival heights were tested and the quality of ocular pursuit was characterized by means of several timing and accuracy parameters. Catching performance differed across subjects and depended on ball flight characteristics. All subjects showed a similar sequence of eye movement events and a similar modulation of the timing of these events in relation to the characteristics of the ball trajectory. On a trial-by-trial basis there was a significant relationship only between pursuit duration and catching performance, confirming that keeping the eyes on the ball longer increases catching success probability. Ocular pursuit parameters values and their dependence on flight conditions as well as the eye and head contributions to gaze shift differed across subjects. However, the observed average individual ocular behavior and the eye-head coordination patterns were not directly related to the individual catching performance. These results suggest that several oculomotor strategies may be used to gather information on ball motion, and that factors unrelated to eye movements may underlie the observed differences in interceptive performance.

## Introduction

Vision is a major source of information for fast and accurate interceptive movements like catching or hitting a flying ball. By bringing and keeping the image of a moving target on the fovea, eye movements allow gathering salient information on the ball trajectory from high acuity vision of its spatial landmarks. Such information is often critical for the control of interceptive task [1–5]. A growing body of studies indicates that the eyes and the hand can be guided by common sources of visual information on the properties of the ball trajectory such as speed, angular velocity, distance, rate of expansion, and, if the ball bounces, as in cricket or tennis, the angle of incidence [5–8]. Likewise, the same representation of the object motion is thought to underlie the control of both effectors [5, 6, 9]. The picture emerging from these studies is that targeted eye movements often precede hand movements and subserve their control. It is then unsurprising that the quality of vision can influence ball catching performance. For instance, if vision is disrupted by occlusion of parts of the ball trajectory, catching performance deteriorates with increasing duration of the occlusion [10–13]. Because of substantial sensorimotor processing delays, tracking fast targets is not always possible [14, 15] and corrective saccades and peripheral vision are often exploited to overcome this limit and pick up relevant information on the ball trajectory [1, 16, 17]. Notice that processing of target information in peripheral vision may be misleading as it alters the perceived speed of the object and can introduce movement biases [18, 19]. In fact, several studies have reported decreasing catching performance with increasing ball speed [20–22].

How vision contributes to the control of the interceptive action is debated [7, 8, 23, 24]. One possibility is that visual cues about the temporal and spatial characteristics of the target trajectory and prior knowledge about the target dynamics allow an accurate extrapolation of its motion. Gaze behavior may reflect the ability to anticipate changes in the visual scene and to predict the future position of the target. Another possibility is that motion execution is updated continuously based on a combination of optical cues (see [8] for a review), guiding the hand toward target interception (so called prospective control). According to this second view, eye movements may provide a measure of the quality of the visual information and efference copy used to guide the action [15, 25, 26]. Several studies have shown that target foveation allows a continuous monitoring of target speed and direction [27, 28], as well as other trajectory features [8]. Such information is crucial for the visual control of the hand [7, 27, 29] and for adjusting the initial motor plan on the way toward the catch zone [27, 28].

Irrespective of the specific strategy which is hypothesized to underlie target interception, it is often assumed that eye movements are informative of the accuracy of the control processes (whether predictive or prospective), and thus are related to the subject's skill in the interceptive task [1, 3]. In line with this expectation, several experiments investigating oculomotor strategies in different ball sports, such as cricket [3, 4, 30], baseball [1], juggling [31–33], volleyball [34], soccer [16], and tennis [35–38], have indeed reported differences in gaze tracking performance across subjects of different expertise. For example, Land and McLeod [3] observed that a key feature of the oculomotor behavior of cricket batsmen is represented by a saccade at the bounce point, which is crucial for the evaluation of the post-bounce trajectory. These authors reported that an expert athlete used the information acquired immediately after ball release to predict the bounce event more accurately and more in advance than an amateur player and a naive player. The expert player was then able to pursue the ball after the bounce longer than the other two players. In another study, professional baseball athletes facing fast ball trajectories kept their eyes on the ball longer and with faster smooth pursuit eye movements than less expert players [1]. Similar differences in gaze behavior between players with different expertise were also observed in volleyball [34].

However, whether accurate gaze tracking of the ball throughout its motion is required to perform successful interceptive movements is still controversial. It is worth noting that the majority of the studies on the relationship between ocular pursuit and interceptive performance have related gaze behavior only to the skill level or the expertise of the subjects, whereas the dependence of hitting/catching success in individual trials on the quality of eye tracking has not been investigated to our knowledge. In fact, there is evidence that ball tracking may vary in duration and quality across subjects of the same skill level [2, 34, 36, 37, 39] or, vice versa, the ability at picking up visual information may not be very different between expert and novice players [36, 40–42]. Moreover, a recent study has shown that several cricket batsmen, taken from a population of 13 sub-elite players of the same skill level, barely foveated the ball, and yet succeeded in the task execution [2]. Similarly, it has been shown that interceptive performance is not impaired if visual acuity is degraded by myopic blur [43]. Others studies reported that novice players attempted to view the ball constantly throughout its trajectory, in contrast with expert players [35]. Recently, it has also been observed that the exact targeting of the bounce point in cricket is not mandatory for the extraction of post-bounce ball flight characteristics, if memory from previous trials or expectation of the physical properties of the ball are also exploited [4]. In summary, these contrasting results suggest that the role of accurate ball tracking for successful completion of interceptive tasks needs further investigation.

In the present study, we explored whether keeping the eyes on the ball is crucial for good performance in a one-handed catching task. In particular, we investigated the relationship between the quality of visual tracking and catching success on a trial-by-trial basis. Nine subjects were asked to catch a flying ball projected from a distance of 6 m and arriving at a reachable position without bouncing on the floor. Thus, our task did not require accurate prediction of the bounce point as observed in cricket or tennis [30, 35, 36, 44]. Different ball flight conditions specified in terms of flight duration and ball arrival height were obtained by setting different ball initial velocities with a custom made launching system [45]. This approach allowed testing ball trajectories which involved different levels of catching and tracking difficulty. In fact, the flight conditions with the highest ball speeds were quite challenging for catching and tracking [1, 22, 46]. The quality of ocular pursuit was characterized by means of several timing and accuracy parameters. Moreover, because it has been recently shown that elite cricket batters couple head rotation to ball movement [30], we also investigated the relative role of the eye and head contribution to ball tracking. Finally, because our previous study of a similar catching task had revealed a large inter-individual variability in arm kinematics [20], we wondered whether such variability could be due to differences in the subject-specific ability at gathering and using salient environmental information sources. Here we expanded and complemented this analysis by also examining whether catching performance was related to different individual gaze tracking behaviors.

## Materials and Methods

### Participants

Nine right handed subjects (6 males and 3 females, labeled S_1_ to S_9_), between 22 and 42 years old (30 ± 6, mean ± SD) participated in the study. Two of them were the first and last author of the study (S_6_ and S_8_ respectively). All subjects had normal or corrected to normal vision, were informed about the procedure and the aims of the study, which was approved by the Ethical Review Board of the Santa Lucia Foundation, and gave their written informed consent to participate to the experiment.

### The task and apparatus

**Fig 1:**
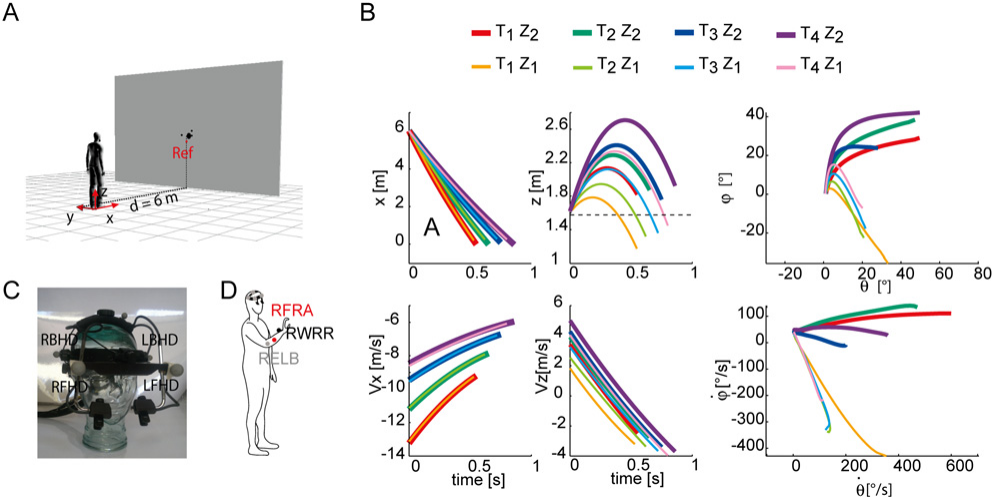
Experimental apparatus, ball trajectories, and marker placement. (A) Subjects were standing at a distance of 6 m in front of a screen with a hole through which balls were projected by a launching apparatus positioned behind the screen. (B) Ball trajectories in spatial and in gaze coordinates for the different experimental conditions examined in the present study. The different T-Z condition are shown in different colors. Solid thick lines represent the Z_2_ conditions, thin lines represents Z_1_ conditions. Top Panels: horizontal (x) and vertical (z) spatial coordinates over time of the average ball trajectory for each block, and the corresponding azimuth and elevation gaze coordinates (rightmost column); the dashed line in the z(t) plot represents the subject eyes height. Bottom Panels: horizontal (x) and vertical (z) spatial velocities of the average ball trajectory for each block, and the corresponding azimuth versus elevation gaze velocities (right most column). (C) Vicon markers placement on the Eyelink helmet. (D) Vicon markers placement on the subject upper trunk.

The experimental task was similar to the one reported in a previous study [20]. Briefly, participants were asked to catch a ball projected by means of a dedicated launching apparatus [45] while standing at a distance d = 6 m (Fig. 1 A). Eight different ball flight conditions obtained by the combination of four mean flight durations (T_1_ = 0.55 s, T_2_ = 0.65 s, T_3_ = 0.75s, T_4_ = 0.85 s) and two mean arrival heights, Z_1_ (low ball arrival height) and Z_2_ (high ball arrival height) were tested. In particular, nominal Z_1_ and Z_2_ were adjusted according to the shoulder height of the subject (H_sh_): *Z*_1_ = *H*_*sh*_-0.3m, *Z*_1_ = *H*_*sh*_ + 0.3m.

The different ball trajectories resulted from a combination of different initial values of the vertical and horizontal velocities and corresponded to different azimuth and elevation angles and angular velocities throughout the flight. Fig. 1B shows elevation angles and angular velocity traces in each flight condition, illustrating the ideal gaze paths of a stationary catcher tracking the approaching ball accurately. In the Z_1_ conditions, ball elevation angle first increased and then decreased. In the Z_2_ conditions, instead, as the ball landed behind the subject, elevation angle increased throughout the entire flight. Ball speed ranged within the [9–14] m/s interval. These ranges have already been tested in similar previous studies [21, 22] and they have been found to be demanding for the perceptual-motor system. Vision of the launcher orientation was impeded by a large big screen in front of the launching machine [45], hence preventing anticipation of the initial ball velocity. Finally, as several sources of random variability affected the ball acceleration and release in our launching apparatus [45], the initial velocity of the ball slightly varied across trials in the same T-Z condition. Thus, in our setup the ball arrival height had a standard deviation of approximately 20 cm [45].

### Data acquisition

Prior to the beginning of the experimental session, the launching apparatus was calibrated according to a procedure described in a previous report [45]. During the experiment, the eye pupil position was recorded at 250 Hz using a head-mounted video-based binocular eye tracker (EyeLink II, SR Research, Ltd., Mississauga, Ontario, Canada). The spatial position of several markers placed on the subject’s body and head, and the position of the ball throughout its entire flight were tracked at 100 Hz using a motion capture system (Vicon-612, Vicon Motion Systems Ltd. UK). Head markers were attached on the EyeLink helmet as reported in Fig. 1C: left front head (LFHD, i.e. M_3_), left back head (LBHD), right front head (RFHD, i.e. M_2_), and right back head (RBHD, i.e. M_1_). Markers were also positioned on the skin overlying the epicondylus lateralis (RELB) and on the right forearm (RFRA) as shown in Fig. 1D. The wrist position (RW) was estimated by averaging the position of two additional markers (RWRU, RWRR), placed at the extremity of a stick (21 cm) taped on the subject wrist in correspondence to the mid-point between the ulnar styloid and radial styloid processes. In addition, the coordinates of the center of the launcher hole and the orientation of the screen were also estimated by means of three markers placed on the screen surface (Fig. 1A). Markers coordinates were referred to a right handed calibration frame placed on the floor at 6 m distance from the launch plane (i.e. the *world coordinate frame*), as shown in Fig. 1A. A consumer-grade PAL mini-DV video camera was used to film the subjects during the entire experimental session.

### Experimental protocol

For each flight condition subjects performed one block of 10 trials, for a total of 8 blocks. If the ball accidentally touched the ceiling of the laboratory or the boundaries of the exit hole on the screen, the launch was repeated. The order of the blocks was randomized across subjects. Prior to the beginning of each session, a few launches with different initial conditions were carried out for task familiarization. Each trial started with an auditory cue to alert the subject of a new launch. After a time interval randomly varying between 1 and 5 seconds to avoid auditory anticipation, the experimenter inserted the ball inside the launching machine and the ball was launched.

Gaze orientation in space was estimated by combining the measurement of eye orientation in the head provided by the eye tracker, and the measurement of head position and orientation in space obtained with the Vicon motion capture system. The detailed description of the procedure is reported elsewhere [47]. Prior to the beginning of the launch session, subjects performed a calibration session required for the extraction of the geometrical parameters of the mapping between the pupil image, as measured by the eye tracker, and gaze coordinates in space. Subjects were asked to gaze at a Vicon marker located on the edge of a stick that was slowly moved by the experimenter within the subject’s field of view. The procedure was repeated at the beginning and at the end of the catching experiment. Additionally, to prevent gaze error estimation caused by accidental slippages of the tracker over the subject head, a drift correction procedure was applied [47]. With the exception of S_4_ and S_7_, all other subjects performed a drift correction trial at the end of each block, which consisted in fixating a marker located on the bottom border of the exit hole of the launching system (i.e. the REF marker in Fig. 1A). The recorded data were then used to estimate the slippage, and hence to correct the geometrical parameters extracted with the initial calibration of the system. In the case of S_4_ and S_7_ the correction was carried out by assuming that the subjects were looking at the exit hole of the launching system when the trial started.

### Data analysis

Subject's catching performance was evaluated considering the percentage of successful catches. In particular, each trial was classified as caught if the ball was captured by the hand and non-caught otherwise. Kinematic data from the Vicon markers were digitally low-passed filtered (25 Hz cutoff frequency, zero-lag FIR filter) and differentiated to obtain the first- and second-order derivatives. As in a previous study [20], ball flight events were characterized by means of several parameters. Ball launch time was defined as the instant at which the ball passed through the screen. Impact time was computed as the instant at which the distance between the ball trajectory and the plane passing for the RWRU, RWRR and RFRA markers reached its minimum. Flight duration was defined as the time interval between the launch time and the impact time.

A trial was not included in the analysis if: 1) the subject initiated the movement before the ball was projected in space by the launching system (2.2 ± 1.3, mean ± SD, trials excluded within the entire session, averaged across subjects); 2) the position of the markers on the head was too noisy or was not reconstructed by the Vicon system for too many frames to allow the Vicon software to accurately interpolate its trajectory (10.7 ± 4.6 trials); 3) the ball was not reconstructed (2.7 ± 1.4 trials) because it exceeded the tracking volume of the Vicon system (i.e. about 7.5m×3m×3m in our case).

### Gaze, eye, and head coordinates

The calibration parameters were used to extract the gaze-in-space orientation angles from the EyeLink-II and the Vicon head markers recordings. In addition, a diagnostic analysis was carried out for each trial to assess which drift correction trial between those recorded respectively at the beginning and at the end of each block gave the best reconstruction outcomes. This procedure ensured high accuracy and precision in the estimation of gaze angles throughout the entire experiment. In particular, for this experiment the estimation of azimuth and elevation angles had an accuracy of 0.07° on average, always better than 0.39°, and a precision of 0.49° average, and always better than 0.80°.

In the following, we will refer to *gaze* and *eye* coordinates as the azimuth (θ) and elevation (φ) angles expressed: a) with respect to the world coordinate frame centered in the eye (i.e. gaze coordinates: θ and φ); b) with respect to a head-fixed coordinate frame system centered in the eye (i.e. eye coordinates: θ_e_ and φ_e_) [47]. The *head* coordinates are the azimuth and elevation angles expressed with respect to the head pose defined while the subject looks straight ahead at a far target at eye height (*the primary position*) by means of the position of three non-collinear points on the helmet (i.e head coordinates θ_h_ and φ_h_).

In brief, eye coordinates express the rotation of the eye inside the orbit, head coordinates express the head position and orientation in space, and gaze coordinates take into account the head position and orientation in space and express the gaze shift with respect to the *primary position*. In particular:

$$\theta =\theta_{e}+\theta_{h},\;\varphi =\varphi_{e}+\varphi_{h}$$ (1)

The position of the ball, tracked with the Vicon motion capture system, was also converted in *gaze coordinates* (i.e., θ_b_ and φ_b_). These angles represent the ideal gaze orientation that a catcher would have if he/she was perfectly tracking the ball.

Different filter cut-off frequencies were applied to the EyeLink-II data depending on the specific analysis being performed. For the purpose of extracting calibration and correction parameters for the estimation of gaze orientation in space, pupil coordinates recorded with the EyeLink-II system and positions of the target and head markers collected with the Vicon system were digitally low-pass filtered respectively at 25 Hz and 15 Hz cutoff frequency (zero-lag FIR filter) as specified in [47]. For the purpose of the saccade identification and analysis, the EyeLink-II raw data were filtered with a 50 Hz cutoff frequency. The calibration parameters were used to compute gaze in eye coordinates. Data were then differentiated to obtain the first- and second-order derivatives. Saccades were identified, using the left eye coordinates, based on a combination of threshold criteria for acceleration and velocity as specified in [48]. Briefly, points in the gaze acceleration trace exceeding a threshold of 1500°/s^2^ were first identified, and the complete saccadic trajectory was determined by finding the peaks and troughs of the acceleration within the interval in which the threshold criteria was exceeded. If the use of the acceleration threshold failed to identify a saccade that could be detected by visual inspection, a second pass was made in which a velocity threshold of 30°/s was applied. For each trial we quantified the number of saccades and, for each saccade and for both the azimuth and the elevation angles, several parameters were computed. The saccade amplitude (A_S_) was defined as the difference between the final and the initial orientation of the eye. The saccade onset or latency time (LT_S_) corresponded to the time the saccade was initiated (see above) with respect to the time of ball launch. Similar to other studies [49], saccades were divided in two groups: the first saccade was used for the characterization of the initial oculomotor response, while the remaining saccades were used to characterize the pursuit phases [15]. Hereafter, we will refer to the latter group as catch-up saccades. Finally for the purposes of the analysis of gaze tracking features (see below) the EyeLink-II raw data were low pass filtered with a 15Hz cut-off frequency in accordance to [50]. All analyses were performed on the left eye data.

### Gaze tracking features

**Fig 2:**
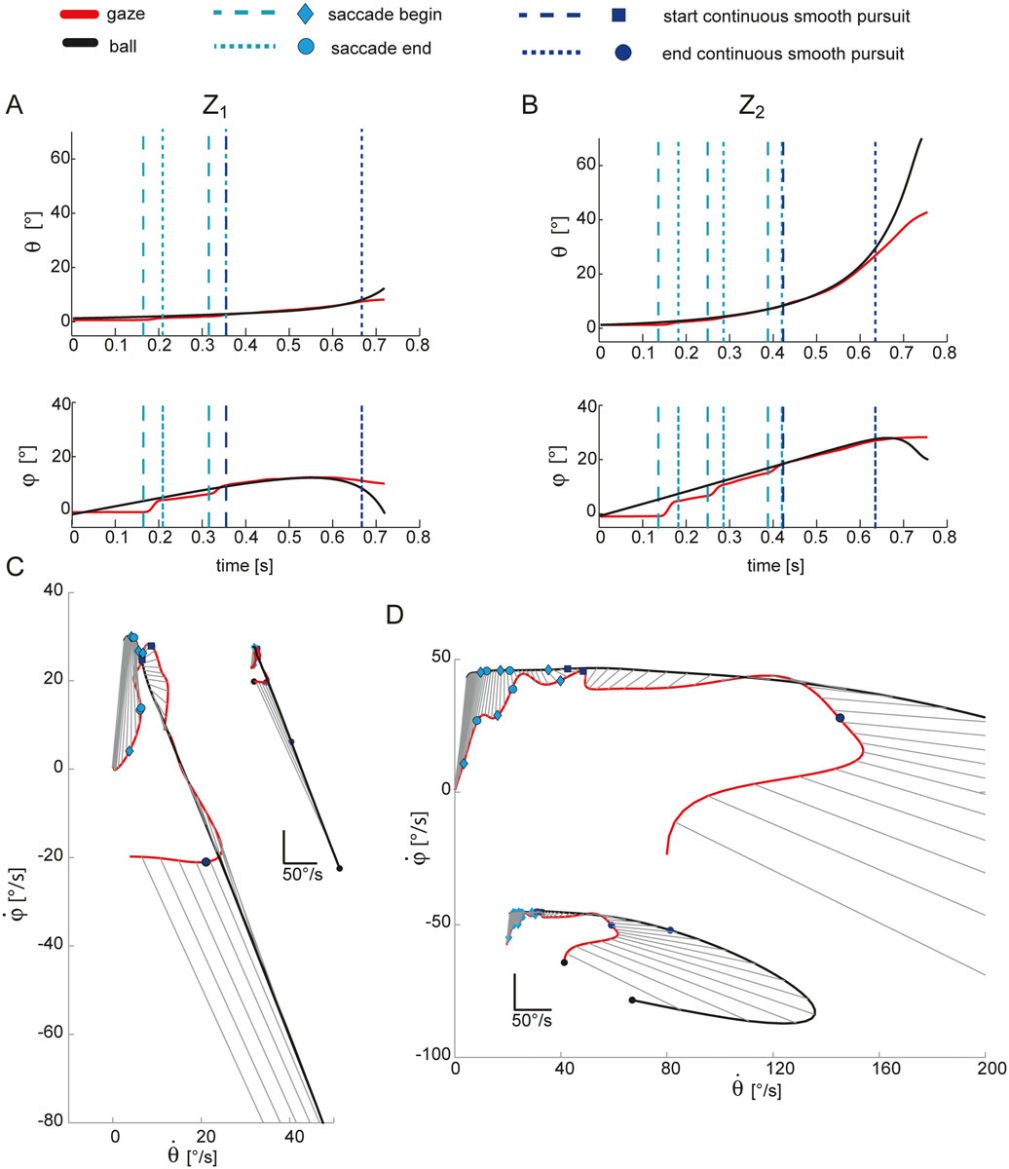
Representative example of eye and ball gaze position and velocity including saccades. (A) Gaze coordinates of the ball (red lines) and the left eye (black lines) are shown for one trial relative to the T_3_Z_1_ condition for one subject (S_9_). Azimuth and elevation trajectories are plotted up to the impact event; cyan vertical dotted lines bound the saccadic intervals and define the catch-up phase tracking; blue dotted lines bound the interval of CT phase; (B) gaze coordinates of the ball and the left eye in on trial in the T_3_Z_2_ condition. (C) scatter plots of the gaze speed in azimuth against gaze speed in elevation for the same trial reported in panel A; (D) scatter plots of the gaze speed in azimuth against gaze speed in elevation for the same trial reported in panel B. Saccadic events are represented by cyan circles (saccade start) and diamond (saccade end); blue square represent the end of the catch-up phase, the magenta circle the end of the smooth pursuit phase, and black circles the impact event.

Representative examples of gaze and ball trajectories are reported in Fig. 2 for one subject (S_9_). Panels A and B show the azimuth and the elevation gaze coordinates of the left eye (*eye*, red lines) and of the ball (*ball*, black lines) in the T_3_ condition, for a low Z_1_ (panel A) and a high Z_2_ (panel B) launch. As in previous studies [3–5, 31, 51, 52], eye movements were characterized by a combination of smooth pursuit and saccades. Thus, the analysis carried out in the present study aimed at characterizing these two types of eye movements and their synergy during visual tracking. To this aim, eye and ball gaze coordinates were extracted using the calibration parameters as specified in [47], and then differentiated. Pursuit quality was evaluated by means of accuracy and timing parameters.

Pursuit accuracy parameters quantified the gaze error during tracking. They included:

- the positional error:
  $$PE(t)=\|{\vec{p}}_{e}(t)-{\vec{p}}_{b}(t)\|$$ (2)
  where ${\vec{p}}_{e}(t)$ and ${\vec{p}}_{b}(t)$are the eye and ball gaze orientation vectors at time *t*, whose components are respectively the horizontal and vertical angles in the (θ, φ) plane.
- the saccadic gain (G_S_), i.e. the actual saccade gaze shift amplitude divided by its desired value, that is the difference between the ball position (expressed in gaze coordinates) at the end and the beginning of the saccadic movement.
  $$G_{s}=\frac{\left\|{\vec{p}}_{e}(LT_{s}+{\text{MT}}_{\text{S}})-\;{\vec{p}}_{e}(LT_{s})\right\|}{\left\|{\vec{p}}_{b}(LT_{s}+{\text{MT}}_{\text{S}})-{\vec{p}}_{b}(LT_{s})\right\|}$$ (3)

Pursuit timing parameters quantified the duration of the different type of pursuit movements (i.e. smooth pursuit and catch-up saccades) throughout the ball flight, and were defined as follows:

- The duration of the catch-up saccade (CUS) phase, i.e. the time interval within the total ball flight duration in which saccades are likely to be triggered during ongoing tracking of the ball. The duration of the CUS phase provides a quantitative measure of the subject's ability at predicting target motion. In fact, catch-up saccades are triggered whenever the positional error accumulates during pursuit because of the lag of the smooth pursuit eye movements with respect to the target motion [15]. They are likely to be present at pursuit initiation [53]. Once the oculomotor system anticipates target motion by predicting its next state, the positional error decreases and hence the probability of observing a catch-up saccade become very low. Thus, the faster subjects are at predicting the current ball motion, the shorter the CUS phase is [15]. We also evaluated the normalized value of the CUS duration, CUSn, obtained by dividing the duration of the CUS phase by the ball flight duration.
- The duration of continuous tracking (CT) phase, i.e. the pursuit time interval in which catch-up saccade generation is inhibited and the ball is foveated continuously. As shown in Fig. 2 the typical eye movement patterns observed in this task include an initial CUS phase in which subjects tracked the ball with a combination of pursuit and catch-up saccade movements, followed by a continuous tracking up to a point where the ball was no longer pursued (no-tracking-phase, NT). Thus, the CT duration was computed by considering the time interval from the end of the CUS up to the last sample in the gaze trajectories before ball impact in which PE < 3°. The dark blue dashed lines in Fig. 2 (top and middle rows) bound this interval.
- The total duration of smooth pursuit (SP), i.e. the duration of the intervals within the CUS and CT intervals in which the target was foveated. Here we assumed that subjects could have followed the ball with an eccentricity of a few degrees to the fovea [15, 25]. Thus, the SP duration was computed eliminating the saccadic movements from the eye movement patterns and then considering the number of samples (i.e. the time interval) in which PE < 3°.

Two additional parameters were used to assess the quality of ball tracking: tracking gain (Gain) and delay (TAU). Tracking gain is often used in behavioral studies of smooth pursuit. Tracking delay quantified the timing accuracy of the pursuit. It is worth noting that the present experiment was carried out in unrestrained head movement conditions. Here, because the velocity of the target was high (Fig. 1) and exceeded the eye velocity saturation threshold (60°/s [1]), ball tracking was achieved with combined eye and head movements. Thus, the analysis was carried out on gaze coordinates instead of eye coordinates. These quality parameters were computed as follows:

- The tracking gain (Gain) was computed as the component of the gaze velocity vector (${\vec{v}}_{e}$) in the direction of the ball velocity vector (${\vec{v}}_{b}$, in gaze coordinates):
  $$Gain=\frac{{\vec{v}}_{e}\cdot {\vec{v}}_{b}}{{\left|{\vec{v}}_{b}\right|}^{2}}$$ (4)
- The time delay (TAU) was defined as the lag of the gaze position with respect to the position of the ball. To evaluate its value we used the same approach reported in [54]. In particular for each time sample *t*, we minimized the error function defined as:
  $$E(\tau )=\left|{\vec{p}}_{e}(t)-{\vec{p}}_{b}(t-\tau )\right|+{\vec{v}}_{e}(t)(1-dir\cos)$$ (5)
  where dircos is the extent to which the gaze velocity was aligned with the velocity of the ball, and it is computed as follows:
  $$dir\cos_{}=\frac{{\vec{v}}_{e}(t)\cdot {\vec{v}}_{b}(t-\tau )}{\left|{\vec{v}}_{e}(t)\cdot {\vec{v}}_{b}(t-\tau )\right|}$$ (6)

We allowed τ to vary within [–200, 200] ms range.

The TAU and Gain pursuit parameters were evaluated during the smooth pursuit time intervals.

### Head contribution to gaze

Head movements were analyzed to assess their contribution to the gaze tracking across subjects and experimental conditions. By definition (see Equation 1), the orientation angles describing gaze in space are the sum of corresponding head and eye orientation angles [47] and any given gaze shift amplitude and direction could be obtained by infinite combinations of the two. We wondered whether the different performance levels observed across our subjects in the catching task could be related to different eye-head coordination strategies as suggested in a recent study of elite cricket players [30]. To this aim, we quantified the fraction of gaze shift due to a change in head orientation:

$$\Delta H_{\vartheta }^{n}=\;\frac{\vartheta_{h}^{\max gaze}-\vartheta_{h}^{launch}}{\vartheta_{}^{\max gaze}-\vartheta_{}^{launch}}$$ (7)

$$\Delta H_{\varphi }^{n}\;=\;\frac{\varphi_{h}^{\max gaze}-\varphi_{h}^{launch}}{\varphi_{}^{\max gaze}-\varphi_{}^{launch}}$$ (8)

where $\vartheta_{h}^{\max gaze}$and$\varphi_{h}^{\max gaze}$ are, respectively, the azimuth and the elevation coordinates in correspondence of the maximum gaze excursion (i.e.$\varphi_{}^{\max gaze}$), while $\vartheta_{h}^{launch}$and$\varphi_{h}^{launch}$ are the azimuth and the elevation coordinates at launch. The closer to 1 is the parameter the higher is its contribution to the gaze. In the case of the eye coordinates,$\Delta E_{\vartheta }^{n}$ and $\Delta E_{\varphi }^{n}$ parameters were computed according to the same expression substituting head with eye coordinates. It is worth noting that the $\Delta E_{\vartheta ,\varphi }^{n}$ and $\Delta H_{\vartheta ,\phi }^{n}$must sum to 1 according to Equation 1.

### Statistical Analysis

A binary response variable Y was used to describe catching success, i.e. *Y*_*ij*_ = 1 if the i-th subject caught the ball at the j-th trial, *Y*_*ij*_ = 0otherwise. The j-th trial was characterized by a specific flight duration (T_k_, k = [1, 2, 3, 4]), ball arrival height (Z_h_, h = [1, 2]), and repetition number within each block.

As a first analysis, the association between catching success and experimental conditions (i.e. 4 flight durations and 2 ball arrival heights) was investigated (TEST 1). To this aim, Generalized Linear Mixed Models (GLMM) were fitted to allow both fixed (i.e. the T-Z experimental conditions) and random (i.e. the subjects) effects to be appropriately accounted [55, 56]. In particular, we implemented the following model:

$$Y_{ij}{}^{*}=\beta_{0}+\beta_{T}t_{j}+\beta_{Z}z_{j}+\varepsilon_{ij}+\mu_{i}$$ (9)

where

- *Y*_*ij*_ * is a latent response variable, assumed to have a Gaussian distribution, linked to the response variable *Y*_*ij*_ for subject *i* in trial *j* through the probit link function, i.e. such that the expected value of the latent variable *E*(*Y*_ij_*) is the inverse of the standard normal cumulative distribution (Φ) of the response probability:
  $$E(Y_{ij}{}^{*})=\;\Phi^{-1}[P(Y_{ij}=1)]$$ (10)
- *ε*_*ij*_ and *μ*_*i*_ are the error terms, which represent respectively the variability within (*ε*_*ij*_) and between subjects (*μ*_*i*_).
- *t*_*j*_ and *z* indicate respectively flight duration and ball arrival height of the j-th trial relatively to the i-th subject; in particular *t*_*j*_ = T_k_, with k = [1, 2, 3, 4] and *z*_*j*_ = Z_h_, with h = [1, 2]. Use of *t*_*j*_ and *z*_*j*_ as categorical variables were also investigated.

A second analysis (TEST 2) was performed to assess the dependence of several pursuit quality parameters on the ball flight characteristics, taking into account differences between individual subjects. Each of the pursuit parameters was considered the dependent variable in a Linear Mixed Model (LMM) [57–59] that takes into account both fixed effects (flight duration and ball arrival height) and random effects (subject). A similar analysis was used in a previous study [20]. As the assessment of which model fits best such random effects structure is a delicate issue [58, 60, 61], we report the complete procedure adopted for each parameter in the Appendix. This analysis allowed testing whether the average values of a parameter varied across subjects and experimental conditions.

We then investigated the relation between catching performance and pursuit quality parameters. As a first explorative analysis, the following model was evaluated:

$$Y_{ij}{}^{*}=\beta_{0}+\beta_{T}t_{j}+\beta_{Z}z_{j}+\beta_{v}v_{ij}+\varepsilon_{ij}+\mu_{i}\;$$ (11)

where the *v*_*ij*_ is the specific parameter under consideration. However, due to the collinearity between the predictors *v*_*ij*_, *t*_*ij*_ and *z*_*ij*_ observed for the majority of the pursuit parameters evaluated in TEST 2 (see the Results section), such model could not reliably assess a dependence of the catching performance on pursuit parameters. Thus, two different models were evaluated (TEST 3 and TEST 4).

We first assessed whether catching performance could be predicted from pursuit quality parameters separately in each T-Z condition (TEST 3). In particular, we wanted to assess both the effect of pursuit quality parameters on the response variable on a trial-by-trial basis, taking into account the different average pursuit parameter of each individual, and the relation between the response variable and the individual average pursuit parameter. Data from all subjects in each experimental condition were fitted using the following Generalized Linear Mixed Model (GLMM):

Yij*=β0+βv(vij−v¯i)+γi(ν¯)i+εij+μi (12)

where *Y*_ij_* is the latent response variable defined above for the i-th subject and for the j-th trial in the T_k_ Z_h_ experimental condition; *v*_*ij*_ is the parameter under consideration, ${\bar{v}}_{i}$is its average value over the trials in the T_k_ Z_h_ condition of the i-th subject, and *ε*_*ij*_ and *μ*_*i*_ are error terms (see above). For each subject the $\left(v_{ij}{}_{}-{\bar{v}}_{i}\right)$ independent variable represents the variation of the parameter from its subject-specific mean in the T_k_ Z_h_ experimental block, and thus it evaluates the relation between the ocular feature ad the catching response once the individual average distribution is taken into account; we will refer to this term as the *variation* term. If the *β*_*v*_ is significant, it means that there are differences in the *v*_*ij*_ value across caught and non-caught trials. The ${\bar{v}}_{i}$ variable instead is used to assess whether the subject-specific average value of the parameter is related to catching performance; we will refer to this term as the *within-subject average term*. If the *γ* coefficient is significant it means that the different catching performances of individual subjects depends on their specific ocular behaviors.

We then further investigated the relation between pursuit quality parameters and catching performance in all experimental conditions after removing the dependence of pursuit parameters on T and Z (TEST 4). Data from all subjects and all experimental conditions were fitted using the following GLMM [62]:

$$Y_{ij}{}^{*}=\beta_{0}+\beta_{vres}r_{ij}+\varepsilon_{ij}+\mu_{i}$$ (13)

where *r*_*ij*_ are the residuals of the mixed model linear regression for the specific feature *v* (TEST 2, see also the Appendix):

$$r_{ij}\;=\;v_{ij}-{\hat{v}}_{ij}$$ (14)

This approach allows to control the partial effect of the variation of *v*_*ij*_ that affects the response variable Y directly. The maximum likelihood estimation was used to fit the mixed models in accordance to [58].

Finally, we investigated whether improvements in the eye tracking and catching performance were achieved later in each block (TEST 5), by extending the TEST 1 and TEST 2 analyses. In particular, we evaluated whether the inclusion of the trial number inside each block as a predictor ameliorates the LMM and GLMM models fit quality. To this aim, we compared the model structure extrapolated in TEST 1 or in TEST 2 separately for each parameter with a second model which included also the trial number parameter (AIC_ntrial_). For instance in the case of TEST 1 we considered the model:

$$Y_{ij}{}^{*}=\beta_{0}+\beta_{T}t_{j}+\beta_{Z}z_{j}+\beta_{ntrial}n_{j}+\varepsilon_{ij}+\mu_{i}$$ (15)

where n_j_ indicates the trial number of the of the j-th trial relatively to the i-th subject. Similarly in the case of TEST 2, we added the *β*_*ntrial*_^*n*^_*j*_ term, to the model that was selected for the specific parameter (see Results section). The two models were then compared according to the AIC test as described in the Appendix. Statistical analyses were performed in the R software environment (R development Core team (2011). R foundation for statistical computing, Vienna. ISBN:3-900051-07-0 URL http://www.Rproject.org) with the *lme4* package (lme4: Linear mixed-effects model using S4 classes. R package version 1.0–5 http://RAN.R-project.org/package=lme4). Multiple comparisons of means (i.e. Tukey Contrasts) were also performed with *multcompare* package.

## Results

### Catching performance differs across subjects and depends on ball flight characteristics

**Fig 3:**
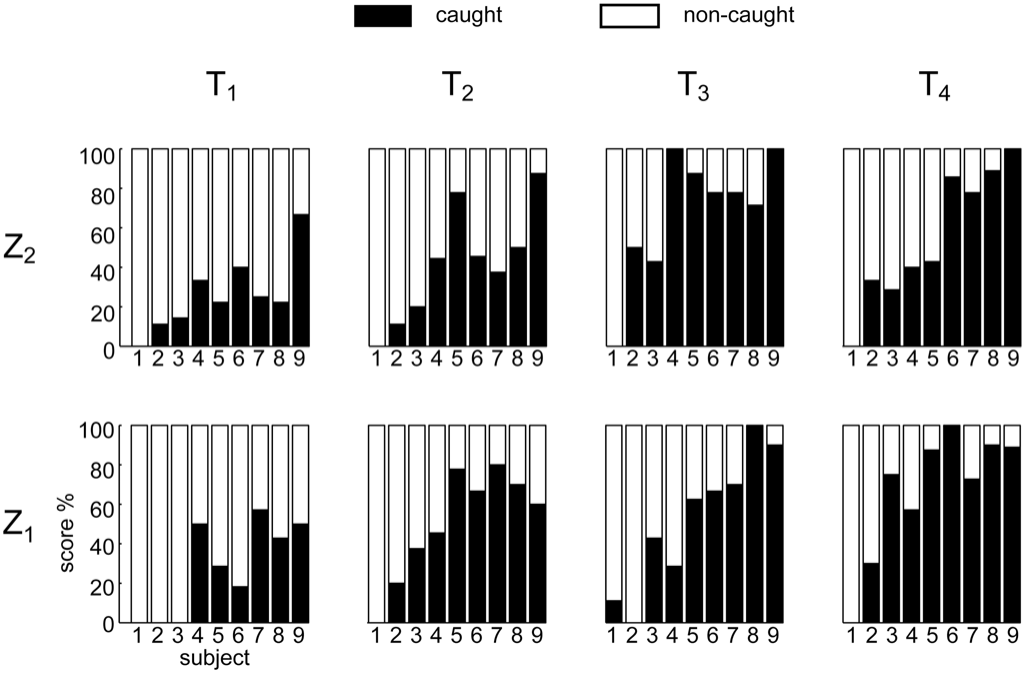
Percentage of caught and non-caught trials in all the experimental T-Z conditions. Subjects are ordered according to their average catching success rate (i.e. the total number of the caught balls divided for total number of launches) from the lowest (i.e. S_1_) to the highest values (S_9_).

The subjects enrolled in the experiment showed broadly varying levels of catching performance. Fig. 3 reports the percentage of caught balls in all the experimental conditions. Subjects were ordered according to their average success rate (i.e. the total number of the caught balls divided by total number of launches), from the lowest (i.e. S_1_, 2% of successful trials) to the highest values (S_9_, 80% of successful trials). For all subjects, catching success depended on ball flight characteristics. The statistical analysis (TEST 1, using GLMM) showed a significant fixed effect of the flight duration T on the response variable Y (Table 1).

**Table 1:** Result from fitted Y* response function for TEST 1 analysis (first row), and from the different eye movement parameters (LMM analysis), for TEST 2.

Parameters	Model	T		Z		T*Z		AIC		R^2^		Random Slope T factor		Random Slope Z factor	
		β_T_	P_T_ ^†^	β_Z_	P_Z_ ^†^	γ_Z_	P_γ_ ^†^	AIC_lmm_	AIC _lm_	Marg.	Cond.	P_S1_	corr	P_S2_	corr
Y*	^§^	5.54	***	0.02	0.68			632.9	765.9	0.06	0.49				
N° Saccades	^†^	3.02	***	0.34	***			1170.8	1283.9	0.23	0.28				
Δφ [°]	**^†††^**	8.69	***	1.71	***			1833.8	2024.7	0.19	0.83	***	-0.98		
LTs [s]	**^††^**	-0.03	0.12	-0.02	**	0.04	**	-3080.4	-2769.5	0.02	0.46				
CUSPn	**^†^**	0.37	***	0.07	***			-737.6	-667.5	0.14	0.28				
SPn	**^††††^**	0.50	***	-0.06	***			-1021.7	-866.8	0.28	0.40			***	-0.75
NTP [s]	^‡^	-0.17	**	-0.06	*	0.09	*	-1902.1	-1902.8	0.04					
Gain	**^†††^**	0.32	**	0.01	*			-832.79	-504.7	0.02	0.80	*	-0.95		
TAU [s]	**^†††^**	-0.01	0.41	0.00	0.76			-2841.8	-2754.9	0.00	0.66	**	-0.92		
ΔH^n^_θ_	**^†^**	0.13	0.62	0.1	0.01			1279.8	1287.8	0.004	0.1				
ΔH^n^_φ_	**^†††^**	0.61	0.012	0.12	***			-238.37	-3.98	0.05	0.78	***	-0.92		

The second column reports the model selected for the specific parameter under analysis (see below for the symbol legend). The subsequent columns report the regression coefficients (β) and p-values (p) of the fixed factors (flight time T, ball arrival height Z) and their interaction term (when significant). The sixth column reports the AIC values computed including the random factor (AIC_lmm_) and without it (AIC_lm_). If AIC_lmm_ < AIC_lm_ the inclusion of the random factor (i.e. subject) is justified, indicating that the particular eye movement parameter varies across subjects. The seventh column reports the marginal and the condition R^2^ coefficient of the regression. Finally the two rightmost columns show the significance of the by-subject adjustment of the slope relatively to both the T factor (p_s1_), and the Z factor (p_s2_), and the correlation between the two random parameters (intercept and slope for each factor).

*: p_value <0.05;

**: p_value<0.01;

***: p_value<0.001.

^§^ (GLMM)Model type Y_ij_* = β_0_ + β_T_t_j_ + β_z_z_j_ + ε_ij_ + μ_i_;

^†^ (LMM) Model type (LMM) v_ij_ = β_0_ + β_T_t_j_ + β_z_z_j_ + S_0i_ +ε_ij_;

^††^ (LMM) Model type: v_ij_ = β_0_ + β_T_t_j_ + β_z_z_j_ + γt_j_z_j_ + S_0i_ +ε_ij_;

^†††^ (LMM) Model type: v_ij_ = β_0_ + (β_T +_ S_li_)t_j +_ β_z_z_j_ + S_0i_ + ε_ij_;

^††††^ (LMM) Model type: v_ij_ = β_0_ + β_T_t_j_ + (β_z +_ S_2i_)z_j_ + S_0i_ +ε_ij_;

^‡^ (LM) Model type: v_ij_ = β_0_ + β_T_t_j_ + β_z_z_j_ + γt_j_z_j_ + ε_ij_

In particular, catching success increased significantly as T increased (T: β_T_ > 0, p_T_ < 0.01), while no significant effect of ball arrival height was found in this data set (Z: p_Z_ = 0.68). Thus, given the relation between flight duration and ball approaching speed (Fig. 1, panel B, first column and second row), success rate decreased as speed increased, in accordance with previous studies [22, 46]. Post-hoc tests revealed that there were significant differences in performance between T_1_ and the other flight duration conditions (p < 0.01 for all the comparison), and between T_2_ and T_4_ conditions. The T_1_ experimental conditions were the most challenging for all subjects: with the exception of S_9_, who showed a success rate slightly below 60%, all other participants caught less than 40% of the balls (Fig. 3). Tracking the ball in these conditions required the highest azimuth and high elevation gaze angular velocities. Notably, S_1_ and S_2_ were unable to catch most of the balls, independently of the speed. In particular S_1_ succeeded only once throughout the entire experiment, while S_2_ succeeded only in 14 trials.

### General characteristics of gaze behavior during catching

**Fig 4:**
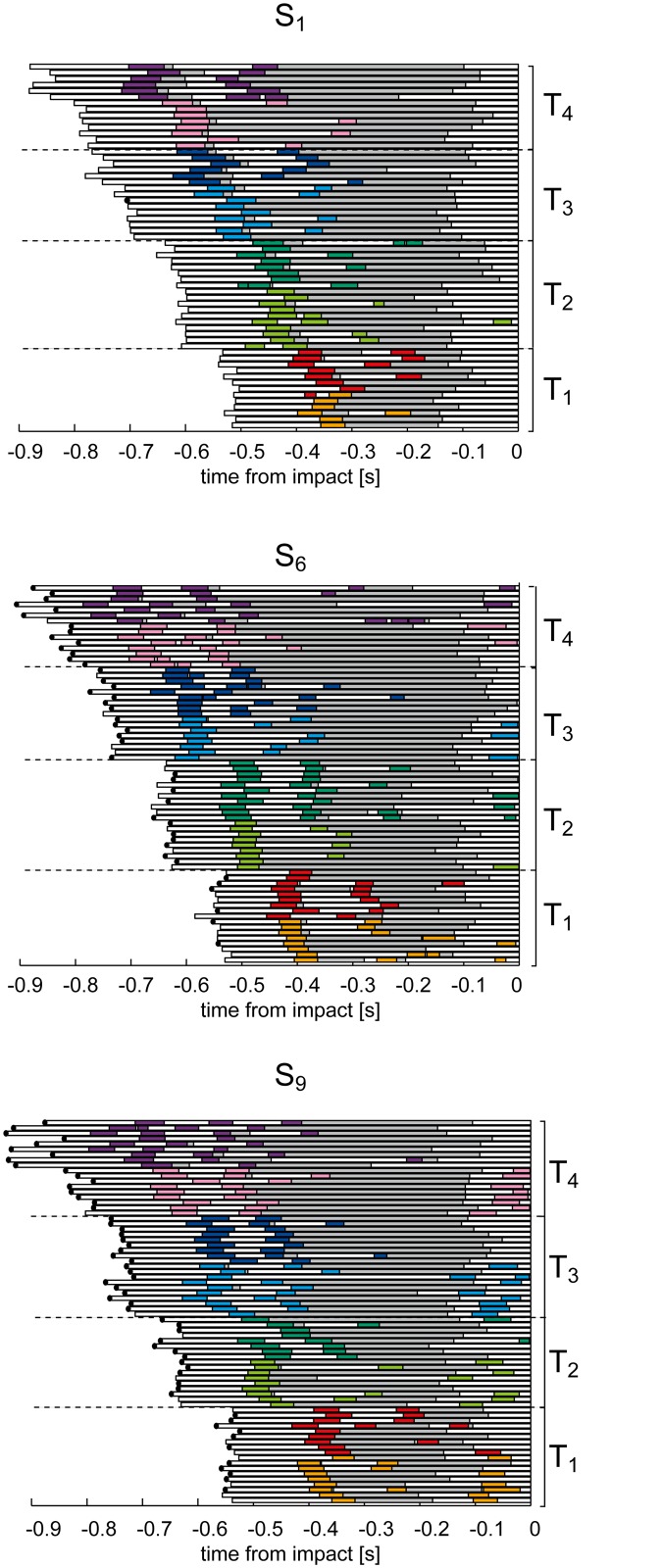
Time course of eye movement events for three subjects of different catching skill level. Raster plots of eye movement timeline are shown for all the trials collected during the experimental sessions. Data are aligned to the impact time. Colored bars represent saccades: the different T-Z conditions are color coded as reported in Fig. 1. The different time conditions are bounded by the dashed black lines. Gray bars represent the intervals in which the target was foveated (i.e. PE < 3°). White bars represent the intervals in which the subject was not tracking the ball. The black dots at the end of each bar indicate caught trials.

All subjects showed a similar sequence of eye movement events. In the first part of the trial, i.e. 30–60% of the total duration, they tracked the ball with a combination of pursuit and catch-up saccades (CUS phase). Afterwards, they continuously tracked the ball without saccades (CT phase), up to a point before impact in which they no longer tracked the ball (*no-tracking*, NT phase). Fig. 4 illustrates all the eye movement events recorded in three subjects (S_1_, S_6_ and S_9_). Each row of the raster plots shows the eye movement timeline in one trial of the experimental session, including saccadic events (*colored bars*, with color identifying different flight conditions), foveal vision intervals (*gray bars*), and periods in which the subject did not track the ball (*white bars*). No-tracking intervals were distributed throughout the flight, and in particular: (1) at the beginning of each trial, while the subject looked at the exit hole of the launching system; (2) between consecutive catch-up saccades, whenever a positional error accumulated during pursuit and exceeded the threshold of 3° (see Methods); (3) at the end of the trial (NT phase), some instants before impact, when additional information on the ball motion presumably could no longer influence hand guidance toward the future impact point [11] due to visual processing and command transmission delays [63]. Occasionally, a saccade was also triggered at the end of the trial prior to impact (see the raster plot for S_9_ in Fig. 4), particularly in the case of low launches. In most cases, subjects re-directed the gaze toward the launching system, while in a few cases they looked the hand at impact.

**Fig 5:**
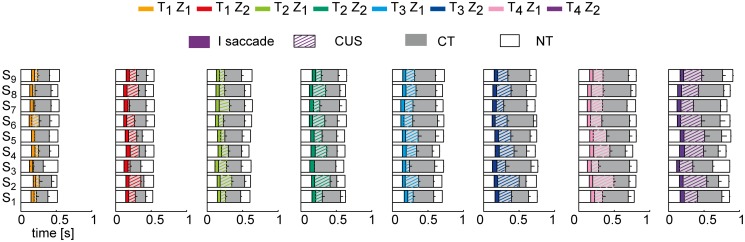
Eye movement event timeline for each subject averaged across trials in all the experimental conditions. Distribution of the eye movement events for each subjects (rows) averaged across trials of each experimental condition (mean ± SE; SE represent by the black lines inside the bars). The different T-Z conditions are reported in different columns. Colored bars represent the average duration of the first saccadic movement; striped colored bars represent the average CUS duration. Gray bars represent the average CT duration. White bars at the beginning and at the end of the raster plots represent respectively the latency time LT_s_ and the NT interval.

These representative subjects give an overview of the general characteristics of gaze behavior observed in this experiment. The average (mean ± SE) values of the timing of different eye movement events in all experimental conditions (columns) is reported for each subject (rows) in Fig. 5. Overall, we observed both differences and similarities in gaze behavior across subjects. Differences were mainly related to the subject-specific distributions of the pursuit quality and timing parameters, and will be described later in this section. Similarities were found in the modulation of the timing of eye movement events (i.e. the first saccade latency, the beginning and the end of CUS and the CT phases) in relation to the ball trajectory characteristics. For instance, in all the subjects as flight duration increased, the duration of the CUS phase (striped colored bars in Fig. 5) and of the CT phase (gray bars) expanded, while the CUS phase was longer and the CT phase shorter in the Z_2_ conditions than in the Z_1_ conditions. We analyze in detail the dependence of the eye movement parameters on the ball flight conditions in the next subsection.

### Gaze behavior depends on ball flight characteristics

**Fig 6:**
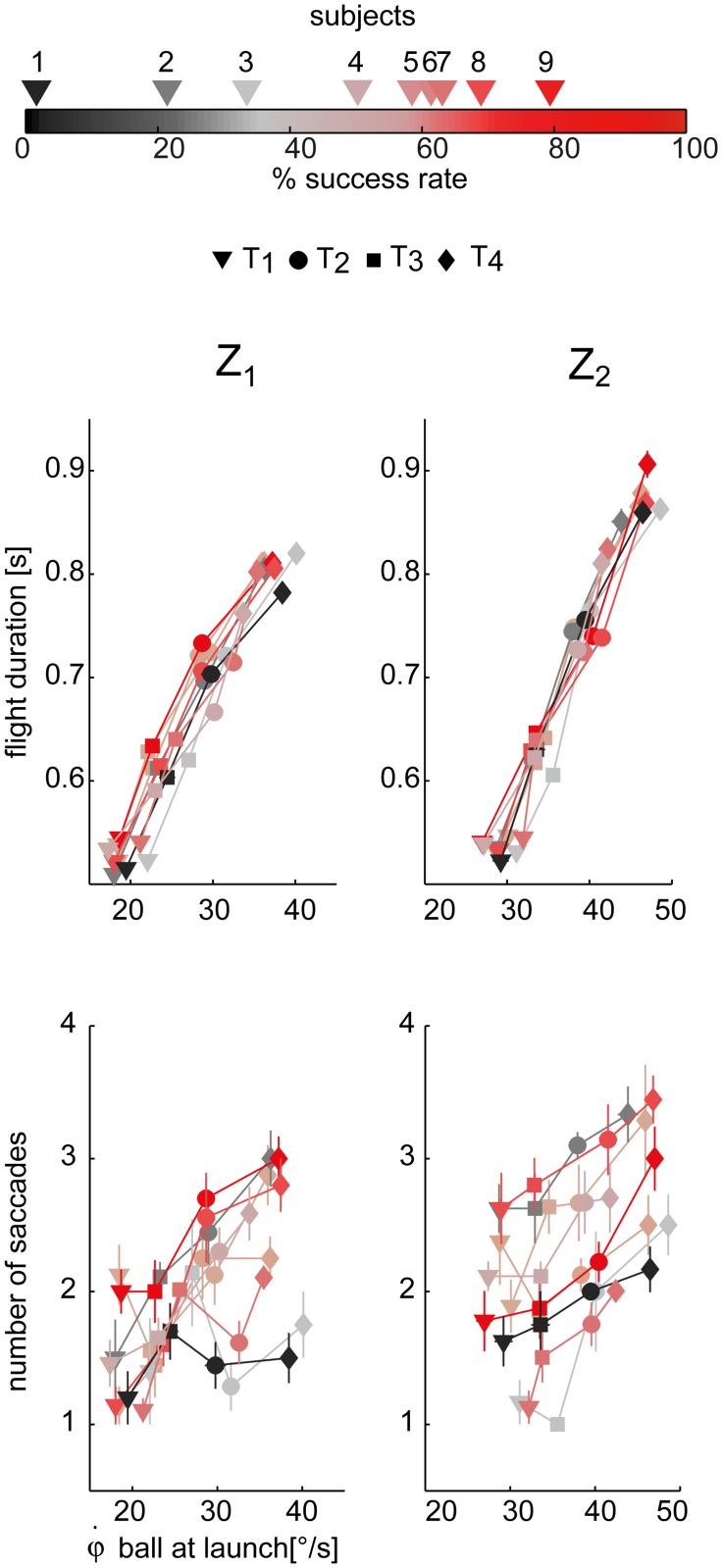
Dependence of ball flight duration and number of saccades on ball initial elevation angular velocity. Top panel: the ball flight duration (i.e. difference between impact and launch time) averaged across the different T-Z conditions (mean ± SE) are plotted with respect to the initial elevation ball velocity at launch. The four different time conditions are indicated by different marker shapes. Data are illustrated separately for the two different arrival heights (right column: high; left column: low). Subject color coding is reported on top of the figure, and it is shaded ranging from black to red depending on subject average score (i.e. the total number of the caught balls total number of launches). Bottom panel: mean number of saccades throughout the trial.

Ball flight duration and arrival height affected both the timing and accuracy of ocular pursuit. In our experiment longer ball flights and higher ball arrival heights corresponded also to higher initial ball elevation velocities, as shown in Fig. 6 (top row) where the relation between flight duration and elevation angular velocity at launch is reported. We performed a LMM analysis (TEST 2) to assess the relationship between eye movement parameters and flight conditions. The results of the analysis of the number of saccades, the latency and amplitude of the first saccade, the duration of CUS and SP phases, the tracking gain and delay are summarized in Table 1.

**Fig 7:**
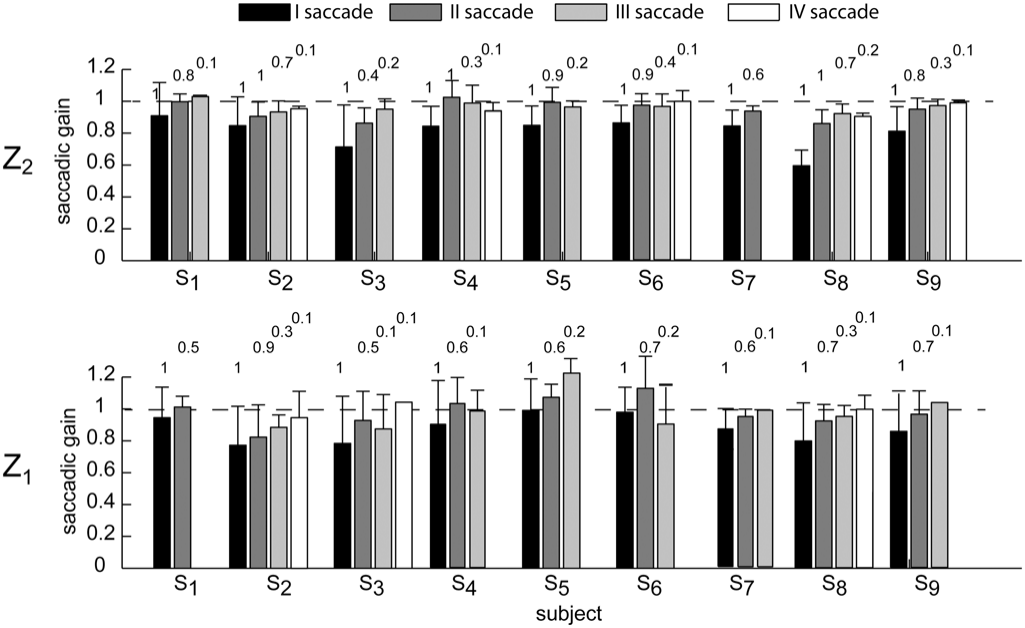
Saccadic gain average distribution across flight duration conditions. The error bar plots show the saccadic gain for respectively the first, the second, the third, and the forth saccade of each subject enrolled in the experiment. Data are averaged across the four flight duration conditions (mean ± SE), and are illustrated separately for the Z_2_ (top row) and Z_1_ (bottom row) ball arrival height conditions. Saccades triggered at end of the trial were excluded from this analysis. The numbers on top of each bar represent the percentage of occurrence of respectively the II, III, and IV saccadic event computed for each subject by pulling together data from the same Z conditions. The first saccade had an occurrence of 100%.

The number of saccades triggered throughout the flight (Fig. 6, bottom panel, data are illustrated separately for the two Z conditions) increased with both flight duration and ball arrival height (β_T_ > 0, β_Z_ > 0, p_T_ < 0.01, p_Z_ < 0.01). Moreover, during the CUS phase, every single catch-up saccade reduced the gaze positional error accumulated during pursuit. In this respect, Fig. 7 shows the average distribution of the saccadic gain across flight duration conditions for each subject, illustrated separately for the two ball arrival heights. On average, the gain increased at each subsequent saccade, being close to 1 at the end of the CUS phase.

**Fig 8:**
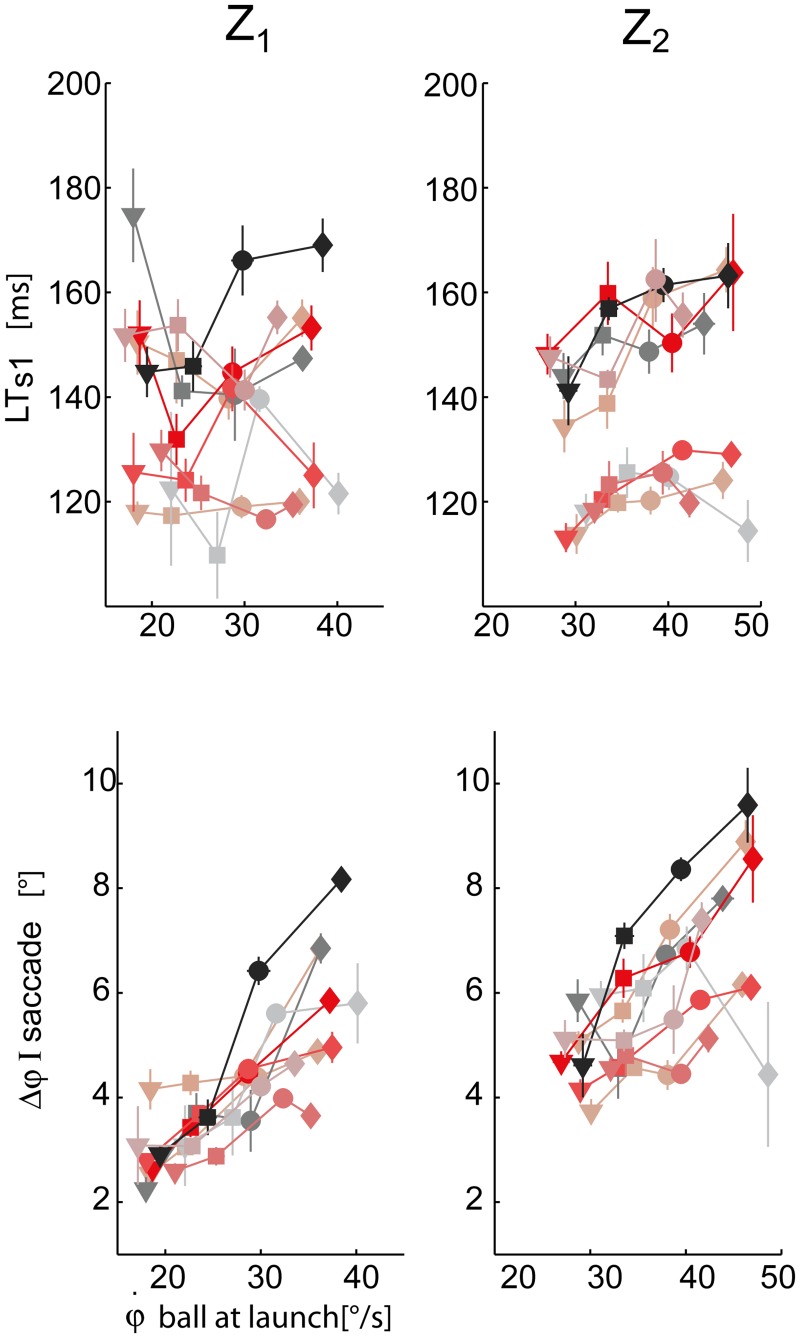
First saccade characteristics. Top panel: first saccade latency times averaged across the different T-Z conditions (mean ± SE) are plotted with respect to the initial vertical angular ball velocity at launch. Data are illustrated separately for the two different arrival heights (right column: Z_2_, left column: Z_1_). The different subjects and the four different flight duration conditions are coded as in Fig. 6. Bottom panel: amplitude of the first saccade.

The initial oculomotor response was characterized by the latency time (LT_s1_) and the amplitude (Δφ) of the first saccade (Fig. 8).

**Fig 9:**
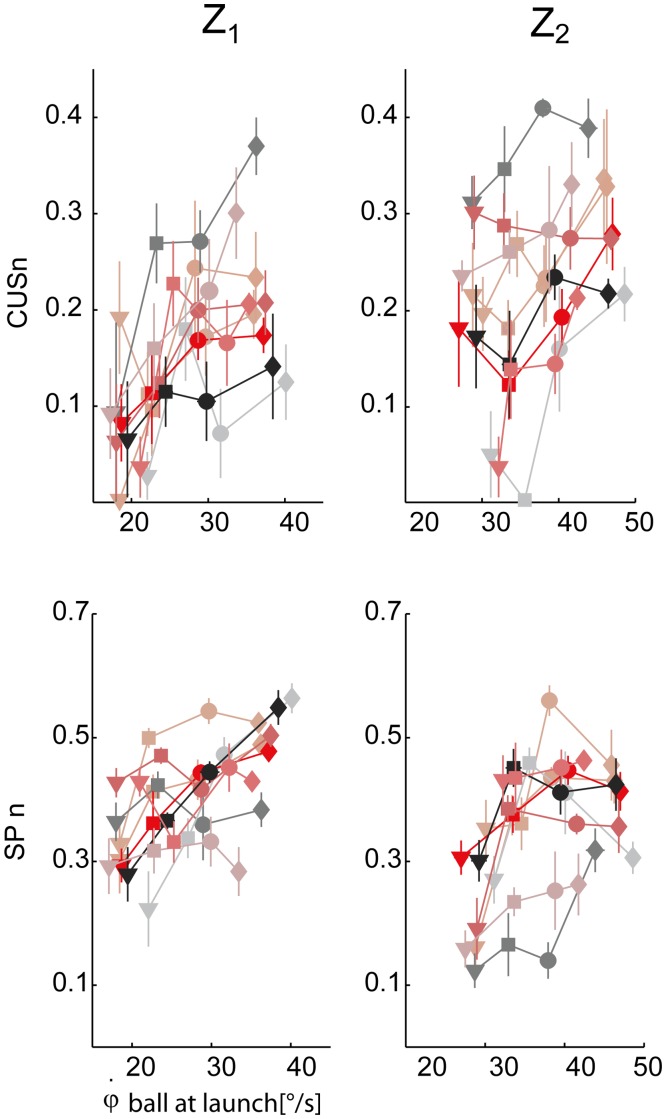
Pursuit timing parameters variation across experimental conditions. Top row: catch-up saccade phase normalized durations (CUSn, mean ± SE for each TZ condition) are plotted with respect to the initial vertical angular ball velocity values. Data are illustrated separately for the two different arrival heights (right column: Z_2_, left column: Z_1_). Bottom row: smooth pursuit normalized duration (SPn). The different subjects and the four different flight duration conditions are coded as in Fig. 6.

**Fig 10:**
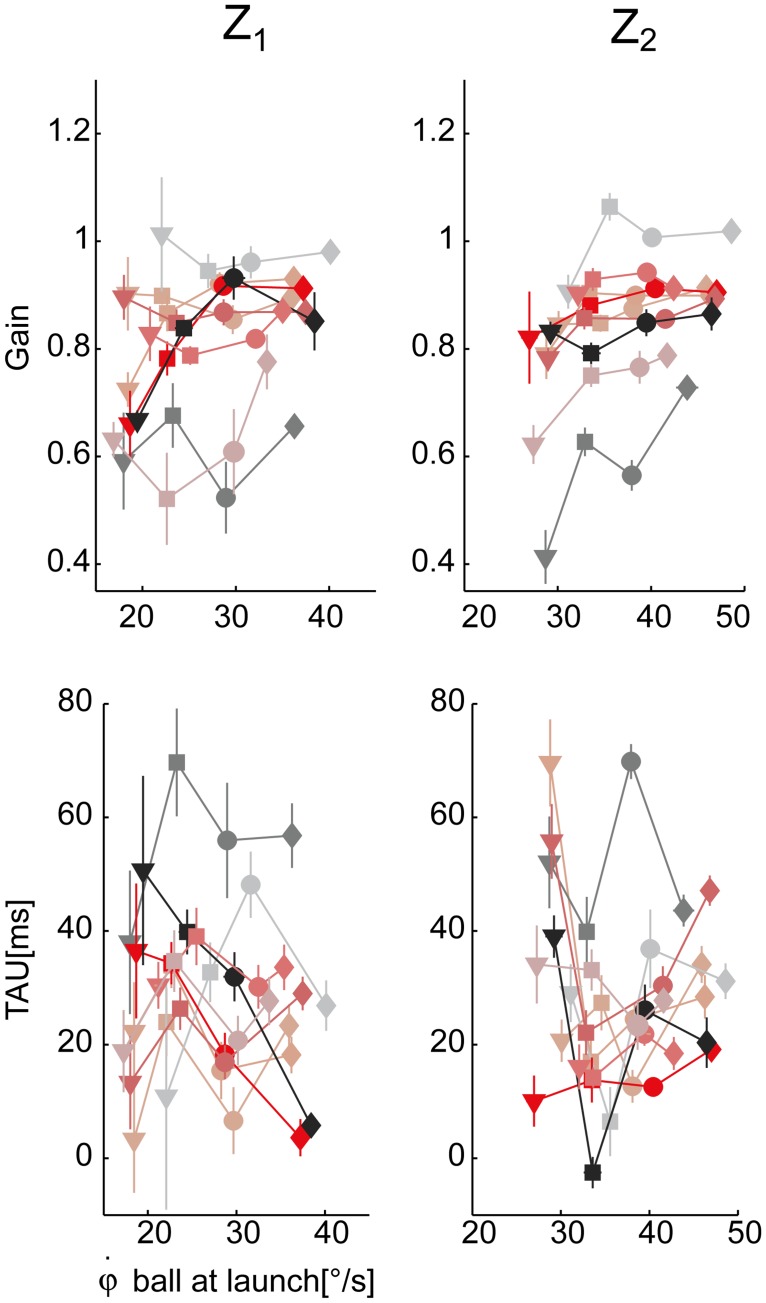
Pursuit accuracy parameters variation across experimental conditions. Top row: smooth pursuit gain parameter averaged for each subject and experimental condition (mean **±** SE) is plotted with respect to the initial vertical angular ball velocity at launch. Data are illustrated separately for the two different arrival heights (right column: Z_2_, left column: Z_1_). Bottom row: the eye-ball positional lag parameter (TAU). The different subjects and the four different flight duration conditions are coded as in Fig. 6.

The interaction term between the T and Z fixed effects was significant (p < 0.01) in the case of LT_s1_. In fact, depending on the ball arrival height, there was a different dependence of LT_s1_ on flight duration. For example, in the Z_2_ condition (high) LT_s1_ increased as the ball flight duration increased, while in the Z_1_ condition (low) LT_s1_ showed a more variable behavior (see Fig. 8 top panels). In the case of high launches subjects appeared to initiate pursuit in advance when facing faster balls (i.e. T_1_ and T_2_ conditions) with respect to slower conditions (i.e. T_3_ and T_4_ conditions). The amplitude of the first saccade was also modulated by ball flight conditions (Fig. 8 bottom panels). Subjects significantly increased the amplitude as flight duration and ball arrival height increased (β_T_ > 0, β_Z_ > 0, both significant). In fact, longer and higher ball trajectories presented also higher initial elevation angular velocities (Fig. 6), which in turn led to larger initial saccadic movements. For this reason data in Figs 8, 9, and 10 are plotted against the initial ball gaze elevation velocity. Differences across subjects were also observed in the distribution of these parameters. They will be described below in a dedicated subsection.

Pursuit performance was characterized by timing and accuracy parameters (see Methods). In order to assess the relationship of the CUS and SP pursuit phases on the ball flight conditions, the statistical analysis (TEST 2) was carried out on their values normalized by the ball flight duration, i.e. the CUSn and the SPn parameters (see Methods). Fig. 9 shows the scatter plot of the CUSn (top panel) and SPn (middle panels) values averaged across the trials of each experimental condition (block) for each subject. The CUSn parameter significantly increased as T and Z values increased (β_T_ > 0, β_Z_ > 0, p_T_ < 0.01, p_z_ < 0.01). Post-hoc tests revealed significant differences between T_1_ and all the other flight time conditions, and between T_2_ and T_4_ conditions. The SPn parameter, which assesses the total duration of continuous tracking throughout the entire flight, significantly increased as flight time increased (β_T_ > 0, p_T_ < 0.01), but it was lower in the case of Z_2_ conditions with respect to Z_1_ (β_Z_ < 0, p_z_ < 0.01). Post-hoc tests revealed that all comparisons were statistically significant, with the exception of the T_2_-T_3_ pair. Overall pursuit duration, given by the sum of the CUS and SP, increased not only because the flight time duration increased, but also because subjects were able to track the ball for a larger fraction of the its flight interval. The quality of tracking during pursuit was assessed by mean of the Gain and the TAU parameters (average values over the SP duration) shown in Fig. 10.

The pursuit gain changed across the ball flight durations, and, in particular, it increased as T increased (β_T_ > 0, p_T_ < 0.01). Post-hoc tests indicated that these differences were mainly ascribed to the T_1_ condition which was significantly different from the others. Significant differences were also observed between low and high launches (p_z_ < 0.05). Gain was higher in low launches than in high launches. Finally, the TAU parameter, i.e. the time lag between the ball and the gaze, was not influenced by the T and Z factors (p_T_ = 0.41, p_Z_ = 0.76).

In summary, in the longest flight duration conditions, the ball was foveated longer and more accurately. The T_1_ conditions were the hardest to track, while on average no significant differences were observed between the T_3_ and T_4_ conditions both in the SPn and CUSn durations and in the gain parameter. These results, together with the observation that also catching performance increased with longer flight durations (TEST 1), may suggest that there is a direct relation between the accuracy and the duration of ball tracking and the subject’s performance in accordance with previous studies [3]. However, both the increase in catching probability and the increase in smooth pursuit duration may be due to a common dependence on ball flight duration (and hence ball speed, Fig. 1) and not to a direct relation between the two variables. To assess the existence of such relationship we considered the dependence on catching performance on pursuit parameters separately in each flight condition (TEST 3) and in all conditions after taking into account the dependence of each parameter on flight conditions (TEST 4).

### Gaze behavior in relation to catching performance

**Fig 11:**
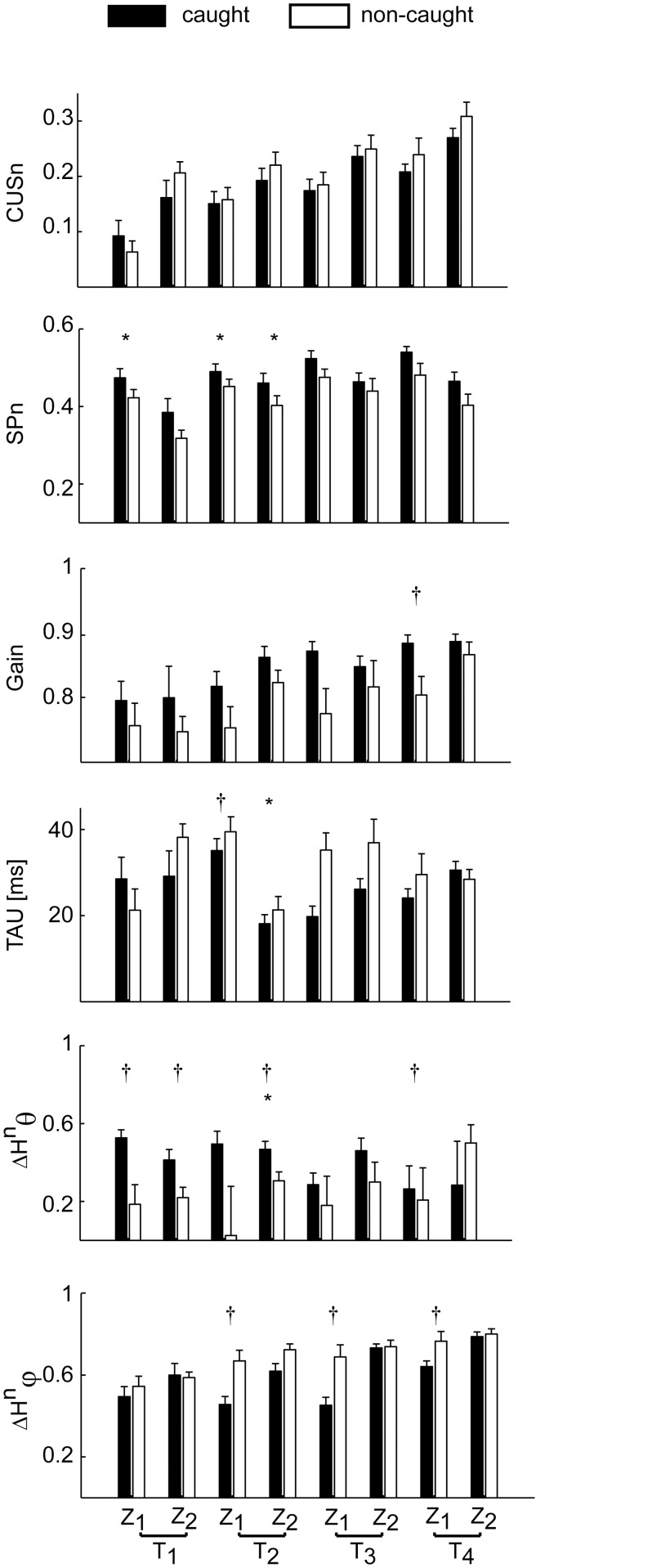
Eye movement parameters averages in caught and non-caught trials. The distribution (mean ± SE across subjects) between caught (black bars) and non-caught (white bars) trials, of respectively CUSn, SPn, Gain, TAU and the head contribution parameters (azimuth θ and elevation φ) is reported for each T-Z experimental condition. The difference between the two score groups are indicated by the asterisks when present. Significance code: * the *variation term* in TEST 3 was significant; † the *within-subject average term* was significant.

To assess the relationship between pursuit parameters and catching performance we first pooled data from all subjects together and we compared the average values of each parameter over all caught and non-caught trials in each flight condition (Fig. 11).

To determine the significance of the resulting differences, we fitted the dependence of the response variable Y* on both the deviation of the value of each parameter in each individual trial from its subject-specific average and on the subject-subject average with a GLMM (TEST 3, see Methods). In fact, differences could be due to variations of the pursuit parameter related to different catching success probability (i.e. the *variation term* in Equation 13) but also to different average values of the parameter in subjects with different average catching performance (i.e. the *within-subject average term* in equation 13). Differences between score groups (i.e. caught and non-caught trials) were not significant for CUSn and Gain in all the eight T-Z experimental conditions (Fig. 11, top panel, leftmost column). Similar results were observed also for TAU (with the exception of the T_2_Z_2_ condition). Significant differences between score groups were observed for SPn in the fastest ball flight conditions. In particular, the normalized duration of smooth pursuit was significantly longer in caught than in non-caught trials in the two T_2_ conditions and in the T_1_Z_1_ condition. Then, to assess whether the relation between the pursuit parameters and the catching performance shown separately on each flight condition (TEST 3) was valid across conditions, after taking into account the subject-specific dependence of each pursuit parameters on the flight conditions, we fitted the dependence of the response variable Y* on the residuals of the regression of the pursuit parameters on the flight conditions (TEST 4). Thus, we tested the effect of the variation of each pursuit parameter on the response variable Y* beside its subject-specific dependence on the ball flight characteristics, evaluated with the analysis carried out in TEST 2. Only the SPn parameter affected the response variable Y* as shown by the significant slope coefficient of the GLMM model (β_SP_ = 5.34, p_SP_ < 0.01). No significant slope coefficients were observed for the rest of the pursuit parameters analyzed in this study.

Overall these results show that catching performance was mainly related to the ability of tracking the ball for a longer time.

### Different pursuit strategies across subjects

The analyses described above showed a relationship between some of the pursuit parameters and both ball flight characteristics and catching performance. For example in TEST 2 we reported that both the SPn, the CUSn, and the Gain parameters presented higher values in the case of the slower flight conditions (T_3_ and T_4_), which were indeed characterized by a larger number of successful trials with respect to the faster conditions (see TEST 1). Next, we tested whether the relationships between pursuit parameters and ball flight conditions (T and Z) significantly differed across individuals. For each parameter, we compared a linear model (LM) relating the pursuit parameter to T and Z for all subjects and a linear mixed model (LMM) including in addition a subject-specific random effect factor (TEST 2, see Methods and Appendix). The models were compared taking into account their complexity with an AIC test: if AIC_lmm_ < AIC_lm_, the inclusion of the random factor was justified providing evidence for significant differences in pursuit strategy across subjects. Moreover, depending on the LMM model selected for the specific pursuit feature under analysis (see Appendix and Table 1), we were able to assess the contribution of the random effect to the inter-individual variability. For instance, a subject-specific intercept in the LMM model indicates that the subjects differs on the average value of a specific pursuit parameter while a subject-specific slope indicates a difference in the dependence of a pursuit parameter on a ball flight parameter (T and Z).

Subject-specific eye movement characteristics were present since the beginning of the pursuit. Significant differences across the subjects were found in the LT_s1_ parameter (AIC_lmm_ < AIC_lm_, Table 1). For instance S_3_, S_6_, S_7_, and S_8_ showed a shorter LT_s1_ than the other subjects (Fig. 8, top row), while S_1_ showed a longer LT_s1_. Inter-individual variability was also observed in the modulation of the amplitude of the first saccade in the different flight conditions. Such difference was captured by an adjustment of the intercept and of the slope of the T factor (p < 0.01) on an individual basis. In particular, S_1_ showed the highest saccade amplitude range (Fig. 8, middle panels). This subject fixated the exit hole of the launching system longer and then triggered a large initial saccade arriving almost on the target as shown by the high values of the saccadic gain for the first saccade in Fig. 7 (black bar). On the contrary, subjects S_8_ presented the smallest saccade amplitude range across subjects. Instead of using a single saccadic displacement, S_8_ triggered a second smaller corrective saccade after the first saccade. In line with this strategy, the saccadic gain distribution reported in Fig. 7 showed that, in the case of S_8_, the gain of the first saccade was much smaller than the gain of the first saccade of the other subjects, while the gain of the second saccade was comparable to the gain of the first saccade of the other subjects. This behavior was particularly evident for high ball arrival heights and long flight durations (i.e. T_2_-Z_2_, T_3_, and T_4_ conditions).

The number of saccades triggered throughout the trial differed significantly among subjects (AIC_lmm_ < AIC_lm_, Table 1). For instance, S_2_, S_4_, S_6_, S_8_, and S_9_ presented a larger number of saccadic events with respect to the rest of participants (Fig. 6 bottom panel). The opposite trend was observed in the case of S_1_ who showed the smallest number instead. In the case of S_8_, this result was related to the presence of the corrective saccades triggered at the beginning of pursuit. In the case of S_9_ and S_6_ in the Z_1_ conditions, it was due to the presence of an additional saccade triggered at time of impact (Fig. 4, bottom and middle panels). Finally in the case of S_2_ and S_4_ the large saccades number was explained by the longer CUS phase duration with respect to the rest of the participants (Figs. 5 and 9).

Marked difference across subjects were found at the level of the CUSn and the SPn parameters (AIC_lmm_ < AIC_lm_, Table 1). However these difference were mainly related to S_2_, S_3_, and S_4._ In particular, S_3_ and S_4_ presented the opposite behavior with respect to S_2_, and showed the smallest CUSn and the highest SPn parameters values (see Fig. 5, bottom panels, and Fig. 9). Notably by repeating the AIC test on a subset of data excluding S_2,_ S3, and S_4_, we found that the random factor was not significant anymore in the case of the SPn parameter (AIC_lmm_ = -611.65 and AIC_lm_ = -620.81).

**Fig 12:**
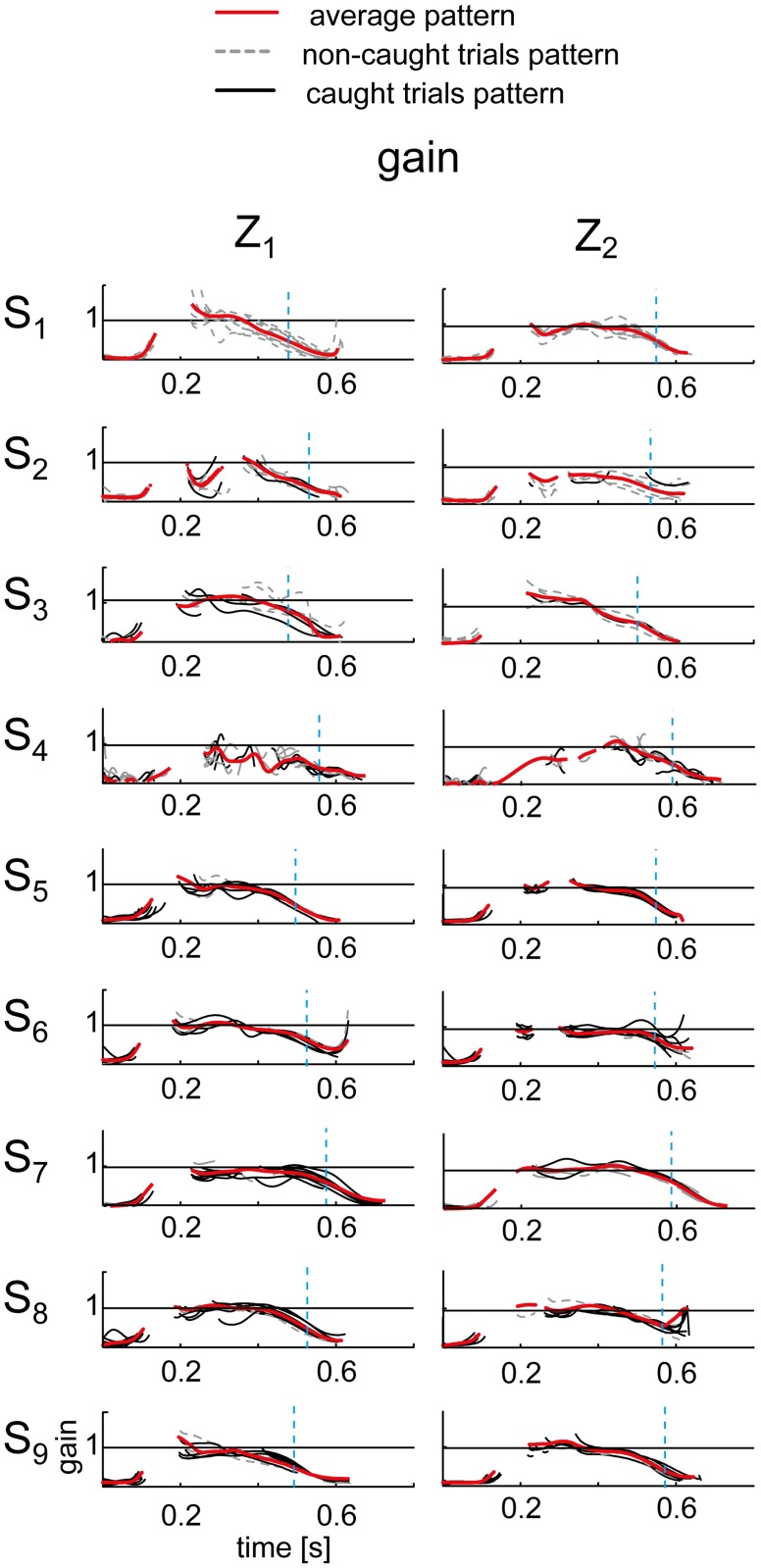
Representative examples of the gain patterns. Data from all the participants and the trials in the T_2_ conditions are shown separately for the two ball arrival conditions. Red lines represent the average patterns across trials. Gray dashed lines represent the non-caught trials pattern. Black lines represent the caught trials pattern. The gaps in the traces represent the occurrence of a saccades in all the trials. Inter-individual variability was mainly attributed to subjects S_2_ and S_3_ who showed on average, respectively, the lowest gain and highest gain values, while all other participants presented similar pursuit gain. Notably, tracking accuracy was similar between the least skilled subject (S_1_) and the best catcher (S_9_). Moreover, no marked differences were visible between caught and non-caught trials.

Further evidence for inter-individual variability was provided by the analysis of the smooth pursuit, the Gain, and the TAU parameters (Table 1 and Figs. 10 and 12). For instance, S_2_ and S_4_ presented the worst tracking performance: the average values of the gain during pursuit were the lowest in all conditions; the TAU parameter, which quantify the eye-ball positional lag, was the highest. Subject S_3_ and S_7_, by contrast, tracked the ball with highest pursuit gain values that in some conditions, i.e. T_1_Z_1_, T_2_Z_2_ and T_2_Z_4_, were on average larger than 1, respectively 1.01 ± 0.23, 1.06 ± 0.05, 1.02 ± 0.03. Subject S_9_ presented poor tracking performance in the T_1_Z_1_ and T_2_Z_1_ conditions as shown by the lower average gain values, i.e. 0.7 ± 0.16. These ranges were comparable with those presented by subjects presenting the worst performance, i.e. S_1_, S_2_ and S_3_, respectively 0.76 ± 0.19 in those experimental conditions (Fig. 10). A representative example of the general patterns of the tracking gain from all the trials and the subjects in the T_2_ conditions is reported in Fig. 12. Finally, according to the AIC criteria, differences across subjects were not significant for the NT duration parameter (AIC_lmm_ > AIC_lm_, Table 1). Subjects stopped looking at the ball on average 0.12 ± 0.05 s (mean ± SD) before impact.

In summary, we found that the dependence of all the pursuit parameters on the flight conditions was different across subjects, indicating the existence of individual pursuit strategies.

### Individual gaze tracking behavior in relation to catching performance

We wondered whether the subjects-specific gaze tracking behaviors described in the previous paragraph were also associated to the individual ability at catching the ball. This information was captured by the *within-subject average term* in TEST 3 (see Methods). We expected that subjects with more accurate gaze tracking presented also higher catching success rates, at least for the SPn parameter in accordance to the outcomes from TEST 3 and TEST 4 reported above.

With the exception of the Gain parameter in the T_4_Z_1_ condition (TEST 3: γ = 13, p_γ_ = 0.025) and the TAU parameter in the T_1_Z_2_ condition (γ = -31.9, p_γ_ = 0.04), the *within-subject average term* in TEST 3 was not significant in the case of the all other pursuit features and ball flight conditions evaluated in this analysis. Hence, the results from the present analysis do not support the hypothesis that superior interceptive performance of a subject is due to his/her superior visual information.

### Effect of practice on catching performance and gaze behavior

As subjects performed blocks of trials with the same ball flight characteristics, we assessed whether the trial number within each block affected the dependence of catching performance and ocular pursuit parameters on flight duration and arrival height. We thus extended the GLMM model of the dependence of the response variable on flight characteristics (TEST 1) and the LMM model of the dependence of ocular pursuit parameters (TEST 2) including the trial number in a block as a fixed effect (TEST 5). The inclusion of the trial number improved the GLMM model fit quality (Table 1, AIC = 632.9, AIC_ntrial_ = 626.8) indicating an effect of practice on catching performance (β_ntrial_ = 0.1 and p_ntrial_ < 0.001). However, repeating the test on subsets of data including only the fastest (i.e. T_1_ and T_2_) or the slowest (i.e.T_3_ and T_4_) ball flight conditions, we observed that the effect of the trial number was significant only in the fastest conditions (T_1_T_2_: p_ntrial_ = 0.006; T_3_T_4:_ p_ntrial_ = 0.246). Interestingly in the case of T_1_ and T_2_ conditions, the significant trial number effect was due only to the two subjects with the best performance (i.e. S_8_ and S_9_). After excluding these subjects the test was not significant anymore (T_1_T_2:_ p_ntrial_ = 0.11).

Ocular pursuit parameters did not change throughout the block. The inclusion of the trial number in the LMM models did not improve fit quality (AIC < AIC_ntrial_) for all ocular pursuit parameters with the exception of the tracking gain (Gain: AIC = -832.79, AIC_ntrial_ = -834.69) which increased within blocks (β_ntrial_ = 0.003, p = 0.04). However, a further analysis revealed that this effect on the gain parameter was ascribed mainly to S_3_. After excluding this subject the test was not significant anymore (Gain: AIC = -758.3, AIC_ntrial_ = -755.0). Finally, the analysis was also repeated including only data from the two fastest conditions (T_1_ and T_2_), i.e. those in which predictive mechanisms were likely to be predominant with respect to on-line control of the movement [63]. In fact, one possibility is that in those cases adaptation in the gaze behavior could have helped improving catching performance (see results of TEST 5 on the GLMM model relative to the T_1_ and T_2_ conditions reported above). For all parameters except the initial saccade latency time (LT_1s_), the inclusion of the trial number did not improve the fit (AIC < AIC_ntrial_). The significant effect for the LT_1s_ parameter (AIC = -1596.8, AIC_ntrial_ = -1599.9) was ascribed only to S_9_. After excluding this subject the test was not significant anymore.

### Eye-head coordination patterns and their relation with catching performance

**Fig 13:**
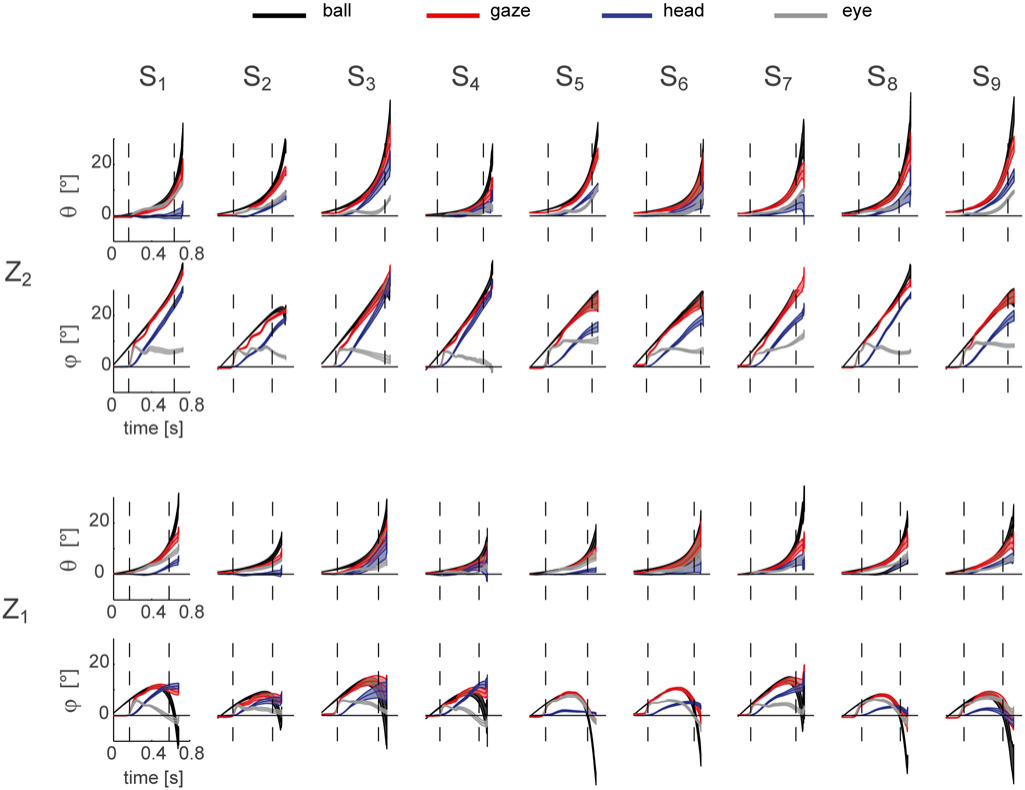
Inter-individual variability in the eye-head coupling. The average (mean ± SE across trials) ball (black lines), gaze (red lines), head (blue lines), and eye (gray lines) azimuth (θ) and the elevation (φ) trajectories in the T_3_ conditions are shown for all the subjects enrolled in the experiment. Top rows: Z_2_ condition; bottom rows; Z_1_ condition. Trajectories are plotted up to the impact event. Dotted lines bound the pursuit interval defined as the time windows between the end of the first saccadic event and the end of the CT phase. For the purpose of figure clarity we keep out the initial offset of both the eye and head position due to small bias with respect to the primary position defined at calibration (see Methods).

As subjects could pursue the flying ball with different combinations of eye and head movements, we wondered if they adopted different eye and head coordination patterns and whether differences in eye-head coordination strategies were related to differences in catching performances. Fig. 13 shows the average head and eye trajectories in the T_3_Z_1_ (bottom panels) and T_3_Z_2_ (top panels) conditions for each subject (mean ± SE across trials). All the participants initiated pursuit with the head still and they used the first saccadic movement to redirect their gaze on the ball. However, different eye-head coordination patterns were observed across subjects and ball flight conditions during the remaining part of the ball flight.

In the case of the elevation coordinate φ in the Z_1_ condition (Fig. 13 forth row), participants showed three different behaviors. S_1_ and S_4_ initiated to counter-rotate the eye inside the orbit (i.e. decreasing the eye elevation angle while the ball elevation angle is increasing) at the end of the first saccade and simultaneously they started to rotate the head towards the ball. This strategy required the eye to rotate in the opposite direction with respect to the head to compensate for a head rotation larger that the gaze shift. In contrast, S_5_, S_8_ and S_9_ pursued the ball mainly with the eye and they either maintained the head almost still (S_5_) or presented very small head excursions (S_8_ and S_9_). Finally, S_2_, S_3_, S_6_ and S_7_ showed a third type of eye-head coordination pattern. At the end of the first saccade they pursued the ball mainly with the head and they maintained the eye at a fixed orientation inside the orbit. In the Z_2_ condition the head contribution to gaze shift was larger than the eye contribution. With the exception of S_3_ and S_4_ who counter-rotated the eye during tracking, and S_7_ who contributed both with the eye and the head to the gaze tracking, the rest of the subjects maintained the eye at a fixed orientation inside the orbit at the end of the CUS phase, and coupled the head to the ball motion (Fig. 13 second row).

Although less marked than for elevation angles, differences across subjects in the eye-head coordination patterns were also present for horizontal gaze tracking, i.e. θ angle, in the Z_1_ condition (Fig. 13 third row). For instance, some subject relied mainly on eye movements to track the ball, i.e. S_1_, S_2_, and S_5_. The rest of the participants instead tracked the ball with a combination of the eye and head movements. This latter strategy was adopted by seven of the nine subjects (S_3_—S_9_) in the Z_2_ condition (Fig. 13 first row), while the remaining two (S_1_ and S_2_) showed larger eye movement than head movements as in the Z_1_ condition.

**Fig 14:**
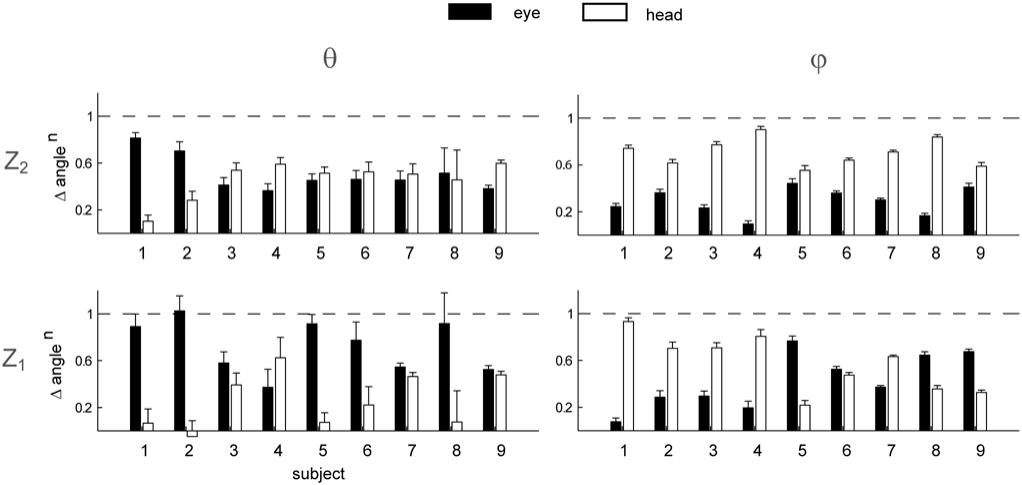
Eye and head contribution to gaze parameter distribution. Left panels: the azimuth eye (i.e. $\Delta E_{\vartheta }^{n}$black bars) and head (i.e. $\Delta H_{\vartheta }^{n}$white bars) contributions to gaze are shown. For each subject data are averaged across the flight duration conditions (mean **±** SE) and they are illustrated separately for the two different arrival heights conditions (top row: Z_2_, bottom row: Z_1_). Right panels: the same is illustrated relatively to the elevation coordinates (i.e. $\Delta E_{\phi }^{n}$and$\Delta H_{\phi }^{n}$).

Similar strategies were also observed in the other flight duration conditions. The statistical analysis (TEST 2) carried out to assess the dependence of the fraction of gaze shift due to head movements on flight conditions (T and Z, fixed effects) and subject (random effect) indicated that differences across participants were significant both for azimuth and elevation angles (AIC_lmm_ < AIC_lm_ for both $\Delta H_{\vartheta ,\phi }^{n}$parameters, see Table 1 where only the results relative to the $\Delta H_{\vartheta ,\phi }^{n}$ parameters are shown, given the complementary relationship with $\Delta E_{\vartheta ,\phi }^{n}$parameters). Fig. 14 illustrates the average values of $\Delta E_{\vartheta ,\phi }^{n}$ and $\Delta H_{\vartheta ,\phi }^{n}$ parameters used to quantify the eye and head contribution to the gaze shift of each subject separately for the high (Z_2_) and low (Z_1_) arrival height conditions (mean ± SE across all flight duration conditions). In the case of vertical gaze angle *φ* and the Z_1_ conditions, S_1_, S_2_, S_3_, S_4_, and S_7_ presented larger $\Delta H_{\phi }^{n}$ values with respect to$\Delta E_{\phi }^{n}$. For S_1_ and S_4_ this was also associated with the smallest $\Delta E_{\phi }^{n}$ values, due to the effect of the counter rotation of the eye inside the orbit (see Fig. 13 in the case of the T_3_ conditions). In contrast, S_5_, S_8_, and S_9_ showed lower$\Delta H_{\phi }^{n}$ values due to their higher eye contribution to gaze shift, whereas S_6_ used a combination of eye and head movement to track the ball. In the Z_2_ conditions, the head contribution was predominant in all subjects (see the large white bars in the top rightmost panels of Fig. 14). Moreover, in accordance to Fig. 13, S_4_ showed the smallest $\Delta E_{\phi }^{n}$values due to eye counter rotation. In the case of horizontal gaze angle *θ* and the Z_1_ conditions S_1_, S_2_, S_5_, S_6_, and S_8_, presented the smallest $\Delta H_{\vartheta }^{n}$values, while the other participants contributed both with eye and head to gaze shift and hence presented similar values of $\Delta E_{\vartheta }^{n}$ and$\Delta H_{\vartheta }^{n}$. A similar contribution of eye and head to gaze shift was observed also in the case of Z_2_ with the exception of S_1_ and S_2_ (see the large black bars in the top leftmost panel of Fig. 14) who tracked the ball horizontally mainly with the eye movements (gray lines in Fig. 13).

We then assessed the relationship between the $\Delta H_{\vartheta ,\phi }^{n}$head contribution parameters and catching performance in each individual flight condition. To this end, first we tested whether the catching performance could be predicted by the $\Delta H_{\vartheta ,\phi }^{n}$ parameter (TEST3). No differences between caught and non-caught trials were found in the case of both $\Delta H_{\vartheta ,\phi }^{n}$ parameters (i.e. the *variation term* β_v:_ in TEST 3: p >0.05), with the exception of the $\Delta H_{\vartheta }^{n}$in the T_2_Z_1_ condition (β_v:_ = 1.23, p = 0.044). Interestingly we observed that in the case of the Z_1_ conditions and the $\Delta H_{\phi }^{n}$parameter, the *within-subject average term*, i.e. γ_S_, was significant in all the flight time conditions except T_1_, and with a negative effect (T_1_Z_1_: γ_S_ = -3.45, p = 0.13; T_2_Z_1_: γ_S_ = -3.86, p = 0.01; T_3_Z_1:_ γ_S_ = -4.28, p = 0.02; T_4_ Z_1_ γ_S_ = -7.8, p = 0.02). According to Fig. 14, the head contribution to the gaze vertical shift was smaller in the case of our most skilled subjects (see the white bars in the rightmost bottom panel). Instead, no significance of the *within-subject average term* was observed for $\Delta H_{\phi }^{n}$ in the Z_2_ conditions. In the case of the horizontal head contribution to gaze parameter, i.e.$\Delta H_{\theta }^{n}$, the *within-subject average term* was significant in four of the total eight conditions, and presented a positive value (T_1_Z_1_: γ_S_ = 3.59, p = 0.02; T_1_Z_2_: γ_S_ = 3.25, p = 0.02; T_2_Z_2:_ γ_S_ = 9.47, p = 0.01; T_4_ Z_1_ γ_S_ = 5.01, p = 0.03). This effect was mainly ascribed to S_1_ and S_2_, the catchers with lower success rates, who indeed presented a larger eye contribution to gaze values than the other subjects (see Fig. 14, black bars in the leftmost panels). It is worth noting however that differences across subjects were less marked than in the case of $\Delta H_{\phi }^{n}$parameter in the Z_1_ conditions. We also checked whether the relationship between catching performance and the $\Delta H_{\vartheta ,\phi }^{n}$ distribution observed in TEST 3 held in all experimental conditions after accounting for the $\Delta H_{\vartheta ,\phi }^{n}$dependence on ball flight conditions (i.e. TEST4). For both $\Delta H_{\vartheta ,\phi }^{n}$parameters the slope coefficient of the GLMM model was not significant (p_ΔH03B8_ = 0.22; p_ΔHφ_ = 0.81), meaning that no differences in the $\Delta H_{\vartheta ,\phi }^{n}$ parameters were present between caught and non-caught trials.

## Discussion

We investigated the extent to which the accuracy in ocular pursuit is related to catching performance. We addressed two different aspects of this issue. On the one hand, we examined whether it was possible to discriminate between caught and non-caught trials according to the characteristics of eye movements, taking into account the fact that different subjects could present different gaze tracking strategies and that both catching and gaze behaviors changed depending on the experimental conditions. On the other hand, we also assessed whether individual average gaze tracking performance influenced the outcome of the catching action. We showed that all subjects presented a similar sequence of oculomotor events and a similar modulation of the timing of these events in relation to the characteristics of the ball trajectory. Gaze tracking performance was better for the slowest ball flight conditions, in which a larger number of successful trials was observed. We also found that keeping the eyes on the moving target for a longer time improved the catching performance on a trial-by-trial basis. However, large differences in ocular behaviors were also observed across our subjects and, differently from what was expected, we did not find a tight coupling between individual gaze tracking performance and catching performance. For instance, the best catchers in our experiment showed an accuracy of ball tracking similar to that of the worst players. These results raise a question as to the role of the visual feedback information underlying the control of an interceptive action. This issue is discussed below.

### Gaze behavior in relation to ball flight characteristics and catching performance

The different ball flight conditions used in our study evoked distinct pursuit responses. When investigating the link between gaze and catching behavior and ball motion characteristics, we observed that, as in several previous studies [1, 22, 64], both gaze and catching deteriorated with increasing ball speed (TEST1 and TEST2). For instance, in the T_1_ and T_2_ conditions, the number of catches decreased (Fig. 3), smooth pursuit lasted less (Fig. 9), and tracking gains values were lower (Fig. 10) compared to the other experimental conditions. In these fastest conditions, there was little time for executing the catching movement, and it is likely that a preplanned movement control policy was preferred to a continuous feedback control policy [8, 65]. In the slowest conditions, instead, ball tracking was less challenging. The subjects pursued the ball for a greater portion of its flight and with a higher accuracy, and showed a higher percentage of successful catches (Fig. 5). In fact, several studies have shown that target foveation improves the perception of its trajectory features [66] and prediction [8, 26, 28].

However, when relating the interceptive performance directly to the gaze tracking accuracy, we observed that only the duration of the smooth pursuit (SPn) affected the catching performance. According to TEST4, when the pursuit lasted longer, the probability of catching the ball was higher. Moreover, the analysis carried out in TEST 3 showed that this was true in almost all the fastest conditions, i.e. T_1_Z_1_, T_2_Z_1_ T_2_Z_2_, given that in these cases the *variation term* was significant. We argue that, in the presence of stringent time constraints, gathering enough visual information for ball motion prediction became a crucial issue, as target foveation could be challenging. It follows that even a small improvement in pursuit duration could be highly beneficial for the interceptive outcome. The lack of a similar relationship in the other conditions could be explained by the fact that tracking was easier at lower ball speeds (i.e. T_3_ and T_4_).

Notably, no statistical differences between caught and non-caught trials were observed at the level of the other pursuit parameters (CUS, Gain, and TAU). For example, we considered the CUS phase duration a quantitative measure of the subject inability at predicting the ball trajectory, and we would have expected higher CUSn values for the non-caught score group (Fig. 11). In fact, catch-up saccades have often been described as a compensatory strategy to reduce the positional error accumulating during gaze tracking [15, 67], rather than a beneficial mechanism for motion prediction as in the case of the smooth pursuit [25, 27, 53]. However, it has been recently proposed that the extra-retinal signals used to control the smooth pursuit eye movement are also involved in the generation of catch-up saccades [68]. Hence the information of the ball motion characteristics could have been gathered not only via an efference copy signal during the smooth pursuit of the ball [26], but also during the pursuit/saccade tracking. In line with this hypothesis, all our subjects showed a marked increase of the saccadic gain at each subsequent saccade in the initial phase of the ball pursuit (Fig. 8).

To our knowledge, this is the first analysis of the relationship between ocular pursuit performance and interceptive performance on a trial-by-trial basis. The present results provided evidence that in our task the performance of an interceptive action is linked to the ability at tracking the ball for a longer time, thus confirming previous findings [1, 3, 26, 27]. In addition, the lack of significant differences observed for Gain, TAU, and CUS features between caught and non-caught trials suggests that the accuracy at keeping the eye on the ball was not critical for catching. Our best subjects (S_6_, S_7_, S_8_, and S_9_) showed tracking gain values within a wide range [0.65–0.98] (maximum and minimum average values within each caught trial observed throughout the experimental session), and time lags within a similarly wide range [1.6–98 ms] during pursuit in the case of caught trials. Thus, the analysis of the gaze tracking accuracy might not be informative of the visual signal processing which subserves motor control execution in our task, as argued by other studies [1, 3, 31, 41]. For instance, information on target motion may be gathered peripherally as suggested in [2, 17]. Recently, it has been proposed that information coming from memory from previous trials or expectation are also used to guide the action [4, 22, 51]. These results could explain previous findings from other groups which reported no decrement in the batting performance of cricket players when the quality of the visual input was perturbed by inducing myopic blur up to +2.00D [43].

### Gaze behavior variability across subjects in relation to catching performance

We observed large differences in both catching performance and gaze strategies across individuals (see Table 1 and Figs. 8, 9, and 10). Subjects differed in the latency of the pursuit initiation, in the amplitude, accuracy, and number of the first saccades, in the tracking duration, pick up information strategies (see the high inter-individual variability in the CUSn and the SPn durations in Fig. 9), as well as in the eye-head coordination patterns (Figs. 12–13). In this context, evidence from previous studies comparing the ocular behaviors of expert and novice players in several ball sports disciplines [1, 3, 30, 42, 69] suggested that that skilled performance relies on superior visual skills. Accordingly, we expected that subjects with a more accurate tracking (i.e. higher gain, longer pursuit time, and shorter lag) also had higher catching success rates.

At single trial level, the logistic regressions analyzed in TEST 4 was significant for the SPn parameter, meaning that longer pursuit durations corresponded to a higher probability of ball catching. At the same time, individual eye movement characteristics were not directly related to individual catching performance level, as the *within-subject average term* in TEST 3 was not significant in all the gaze tracking parameters analyzed in the present study. In fact, the subjects who tracked the ball longer were not necessarily the subjects with the best catching performance. For instance, S_3_ showed the highest tracking gains and smooth pursuit durations but he caught only 31% of the balls. Similarly, S_1_, who succeeded only in 2% of the cases, tracked the ball accurately with values of smooth pursuit durations, saccadic gain, and pursuit gain comparable to those of the best performing subjects (Figs. 4, 9, and 10).

The lack of a direct relationship between the ocular pursuit performance and the subject's catching ability observed in our experiment questions the generality of the conclusions drawn from previous studies investigating visuomotor coordination in interceptive tasks. High-acuity visual information has quite often been considered a prerequisite for good performance in these tasks. Our findings provide evidence that superior visual information is not a necessary condition for superior performance. For instance, more effective use of peripheral vision could explain the differences in the interceptive outcomes observed across the participants of our study. However, other non-visual factors may underlie the lack of a relationship between individual ocular and catching performances observed in our experiment. As suggested above, intercepting a moving target is not only a matter of gathering best visual information. Eye movements might be used to estimate the kinematics of the target, but other factors more related to hand-ball interaction may play a key role for the generation of the required motor plan [70]. These factors may affect the fine tuning of the grasping phase or the generation of the appropriate contact forces at the hand, and may require prior knowledge of the physical properties of the ball, such as its mass [70, 71] or size [8].

We are not suggesting that vision is not important for interception. Rather we argue that its relevance in the generation of the motor responses observed in other studies [3] also depends on the specific task constraints. For instance, discontinuities were not present in the ball paths employed in the present study. Thus, subjects were not forced to gaze at relevant points before the potential change of the ball trajectory as it happens in cricket or in juggling. In contrast, visual information acquired intermittently during the ball flight was likely sufficient to regulate the action and to guide the hand toward the impact point [3, 27, 72]. For this purpose, different gaze behaviors could be similarly effective at gathering the same amount of information on the ball motion, as the inter-individual variability in the oculomotor responses observed in the present study suggests. Recently, it has been shown that in a simulated fly-ball of a baseball video game, when asked to intercept the ball controlling a mouse, subjects were more successful when they gazed for longer periods on the cursor if the ball was visible throughout the entire flight [73]. Instead, with targets occluded near the end, subjects achieved better performance by tracking the target more accurately and by avoiding gaze shifts near interception, suggesting that this target tracking provided better trajectory predictions for the interceptive response [73]. These results further support our claims that the relation between gaze and manual interceptive outcomes depends on the task constraints.

### Eye-head coordination variability across subjects in relation to catching performance

In the present study, we also investigated whether the inter-individual variability observed in pursuit and catching behaviors was related to differences in retinal and extraretinal (i.e. based on proprioception of the neck muscles due to head movements) information pick-up strategies. We characterized the eye and head coordination patterns and in particular their contribution to the total gaze shift. Recently Mann and coworkers [30] showed that professional cricket players directed the gaze to the bat at impact. To this aim, in the post-bounce trajectory of the ball they pursued it mainly with the head. In contrast, less expert players closely aligned their eyes with the ball during the entire flight. It has been suggested that, by coupling the direction of the head movement to the motion of the ball, expert players could more easily derive the arrival position and control the hitting movement in a self-centered coordinate frame. In our experiment, we also observed a large variability in the eye-head movement patterns across subjects (Fig. 13), particularly at the level of the vertical gaze shift, i.e. the $\Delta H_{\phi }^{n}$parameter, in the Z_1_ conditions. Subjects with an overall high performance level tended to track the ball more with the eyes than with the head, and viceversa in the case of the low level subjects. With the exception of the T_1_ condition, the *within-subject average term* in TEST 3 was always statistically significant for the $\Delta H_{\phi }^{n}$ parameter. Surprisingly, the opposite behavior was observed with respect to the horizontal gaze shift, i.e.$\Delta H_{\vartheta }^{n}$. Although the *within-subject average term* in TEST 3 was not significant in some conditions (see the Results section), there was a tendency toward using more the eye than the head for ball tracking in the case of low performance level subjects S_1_ and S_2_ (see the large black bars in the leftmost columns of Fig. 14). It is worth mentioning, however, that in the Z_1_ conditions differences across performance level were less marked than in the case of the $\Delta H_{\phi }^{n}$ parameter, given that also S_5_, S_6_, and S_8_ showed a larger contribution of the eye movement, i.e. the $\Delta E_{\vartheta }^{n}$parameter, to the total gaze shift over the head (see the black bars in the bottom leftmost panels of Fig. 14). Notably, our analysis was carried out separately for the vertical and horizontal gaze angles, because we observed that the azimuth component of gaze becomes large when the ball approaches the subject in the final part of its trajectory (see Figs. 1, 2, and 13), whereas the visual angle or only one direction of the visual excursion, considered as the relevant one, were often evaluated in previous studies [1, 2, 4, 39, 42].

In sum, our results are in line with the findings of Mann and colleagues [30,43] relatively to the horizontal gaze shift, i.e. the $\Delta H_{\vartheta }^{n}$ parameter, but they differ in the vertical direction, i.e. the $\Delta H_{\phi }^{n}$parameter. It is likely that in the case of $\Delta H_{\vartheta }^{n}$ low performance level subjects were less capable at maintaining head-ball coupling due to the large angular velocities observed in the final part of the ball trajectory (see Fig. 1 rightmost panels and Fig. 2). Nevertheless we believe that the different nature of the tasks employed in our study and in the study by Mann and colleagues might explain the different results we obtained for the $\Delta H_{\phi }^{n}$parameter. The peculiar head movement strategies observed in professional players in [30] arose from their necessity to see the ball while hitting it with the bat, and hence these strategies concerned more the final part of the ball trajectory. However, in our experiment, subjects had to catch the ball with the hand. Proprioception instead of visual feedback was likely sufficient for limb positioning and orientation as argued in [74]. Accordingly, our subjects stopped watching the ball ~120 ms prior to contact (i.e. NT phase). Instead, the interaction with the bat in cricket (or a racquet in tennis and squash) is likely to require shifting of the gaze from the moving target to the future ball-bat contact. This mechanism may facilitate fine tuning of the arm and wrist orientation and it would be beneficial for the hitting movement execution as suggested in [30]. Moreover, no discontinuities were present in the ball trajectory in our experiment. While gathering the major amount of visual information in the post bounce is crucial for interception in the case of cricket or tennis sports due to the abrupt change of ball motion direction [3–5], in our experiment the smooth nature of the ball path may have reduced the importance of visual feedback at ball-hand contact for action guidance.

Overall, our results are more in line with other previous findings, which reported flexible behaviors of the eye and the head contributions to gaze both between and within subjects in a similar task [39]. These findings suggest that different information pick-up mechanisms may have been exploited by our subjects to capture information on the target motion.

## Conclusions

We investigated the relation between the catching performance and the gaze tracking accuracy in a one handed catching task. On a trial-by-trial basis, we showed that the ability at tracking the ball longer increased catching probability. At the same time, we could not find a significant relationship between the subjects' average accuracy and duration of the ball visual tracking, on one hand, and the subjects' level of catching performance, on the other hand, as observed in previous studies. Instead, we showed that different gaze behaviors and eye-head coordination strategies may emerge in different individuals. These results suggest that other non-ocular factors underlie the observed variability in the sensorimotor strategies and performance outcomes observed in our experiment. One of the assumptions that has often been made in the neuroscience literature is that there are optimal behavioral and perceptual strategies toward which subjects converge when performing a complex task [75]. On the other hand, it has also been shown that goal equivalent motor control solutions are possible when task constraints are relaxed [20, 76]. Thus, a number of perceptual strategies were probably possible to gather information about ball kinematics in our experiment. For example, subjects could track the ball continuously, but rely on motion signals at some instant or on average signals across presentation time [66]. Also a different use of retinal and extraretinal cues was possible [39]. We argued that subjects’ intention, attention, and inter-individual variability in the sensitivity to different types of visual cues could have played a role in the manifestation of one strategy with respect to the others, while non ocular factors, more related to the hand-ball interaction, probably affected the success of interceptive movements. Future studies will investigate the potential interactions between these different elements in the control of interceptive movements.

## Appendix

The use of the Linear Mixed Model is appropriate whenever the experimental design presents crossed fixed effects, here the different T- Z experimental conditions, and random effects, i.e. the subjects enrolled in the experiment [58, 59]. The first step of our analysis was aimed at assessing whether the inclusion of the random effect in the model, that is the use of LMM instead of a simpler Linear Model LM, was justified. To this aim, we used the same approach described in our previous study [20]. In particular we fitted the two models:

$$LMM_{0}:v_{ij}=\beta_{0}+\beta_{T}t_{j}+\beta_{Z}z_{j}+S_{0i}+\varepsilon_{ij}$$ (1A)

$$LM_{0}:v_{ij}=\beta_{0}+\beta_{T}t_{j}+\beta_{Z}z_{j}+\varepsilon_{ij}$$ (2A)

where *i* is the i-th subject and *j* is the j-th trial, *v*_*ij*_ is the eye movement feature under analysis, and S_0i_ is the offset term that accounts for the by subject adjustment of the intercept term, i.e. the deviation from the *β*_0_. To evaluate the significance of the random effects, that is the presence of inter individual variability in the parameter distribution, we used the Akaike Information Criterion (AIC) for both the LMM and LM analysis [55]. The AIC evaluates the quality of the fit taking into account the complexity of the model (the lower the AIC value, the better the model fitting, see [57]). If the AIC_lmm_ resulted lower than the AIC_lm_ the inclusion of the subject factor was justified, providing evidence for inter-individual differences. The level of significance was set as p <0.05.

The second step consisted in the evaluation of the interaction between the fixed effects. If the previous test confirmed the significance of the random effects inclusion, LMM_0_ was compared with the following model:

$$LMM_{1}:v_{ij}=\beta_{0}+\beta_{T}t_{j}+\beta_{Z}z_{j}+\gamma t_{j}z_{j}+S_{0i}+\varepsilon_{ij}$$ (3A)

Otherwise the simpler linear model was evaluated and no more actions were required:

$$LM_{1}:v_{ij}=\beta_{0}+\beta_{T}t_{j}+\beta_{Z}z_{j}+\gamma t_{j}z_{j}+\varepsilon_{ij}$$ (4A)

Mixed models comparison and the p-values of the slopes coefficients were computed according to the likelihood ratio test as reported in [58]. Once the random and fixed structures were assessed, the resulting model was re-fitted according to the restricted likelihood criteria which is more conservative.

Once the structure of the fixed effect was assessed, the next step was the identification of the random effects structure. For instance, the model could include either a simpler structure that accounts only for between-subjects manipulations, hence introducing only by-subjects adjustments to the intercept as in (16), or a more complex model which also allowed for within-subject manipulations. In this latter case, the extent to which subject's effect deviates from the population fixed effect slopes was also investigated.

In accordance with several dedicated studies [25, 58, 60, 61] we adopted the "confirmatory hypothesis testing" approach to identify the best model that fits our data. Briefly, we identified a set of specific critical hypotheses on the data structure and then measured the evidence for or against them. Typically the models are built iteratively, starting from the simpler model structure and then adding more random factors incrementally. At each iteration the simpler and the more complex models are compared according to the likelihood ratio test as reported in [58].

The models evaluated for the present analysis are listed below in sequential order (in the following we will consider the interaction term not significant):

$$LMM_{2}:v_{ij}=\beta_{0}+\left(\beta_{T}+S_{1i}\right)t_{j}+\beta_{Z}z_{j}+S_{0i}+\varepsilon_{ij}$$ (5A)

where the slope offset S_1i_ captures how much each subject deviates from the slope relative to the ball flight time factor T. By comparing the LMM_2_ and LMM_0_ we assessed both whether the inclusion of the random slope factor was appropriate, and the significance of the S_1i_ coefficient. If this hypothesis was confirmed, LMM_2_ model was compared with the following:

$$LMM_{3}:v_{ij}=\beta_{0}+\left(\beta_{T}+S_{1i}\right)t_{j}+\left(\beta_{Z}+S_{2i}\right)z_{j}+S_{0i}+\varepsilon_{ij}$$ (6A)

where in addition to the S_1i_ offset, the amount each subject deviates from the slope relative to the ball arrival height factor Z was considered by the S_2i_ coefficient. Otherwise LMM_0_ was compared with:

$$LMM_{4}:v_{ij}=\beta_{0}+\beta_{T}t_{j}+\left(\beta_{Z}+S_{2i}\right)z_{j}+S_{0i}+\varepsilon_{ij}$$ (7A)

Table 1 reports the selected models and the outcomes of the analysis for each pursuit parameter. In addition the coefficients of determination were also computed. In particular we separated the contribution of fixed and random effects by calculating both the marginal (i.e. the proportion of variance explained by the fixed factor) and the conditional (i.e. the proportion of variance explained by both the fixed and random factors) *R* ^2^ parameters according to the following formulas:

$$Rsq\_conditional=\;\frac{\sigma_{fix}^{2}}{\sigma_{tot}^{2}}$$ (8A)

$$Rsq\_conditional=\;\frac{\sigma_{fix}^{2}+\sigma_{rand}^{2}}{\sigma_{tot}^{2}}$$ (9A)

## Supporting Information

S1 DataSet A compressed archive (.zip) contains a data file and a text file with a detailed description of the data organization. The data file is in Matlab format (.mat) and contains a data structure with, for each subject and for each trial: 1) the gaze coordinates estimated from the eye-tracker recordings as specified in the Methods; 2) the head, the body, and the ball data recorded with the Vicon Motion capture system; 3) the information about the trial characteristics (i.e. the T—Z condition, the catching score, and the launch and impact time events).
(ZIP)
