# Supplementary material for: Gaze Behavior in One-Handed Catching and Its Relation with Interceptive Performance: What the Eyes Can't Tell
Source: PLoS One. 2015 Mar 20;10(3):e0119445. doi: 10.1371/journal.pone.0119445 (PMC4368737; doi:10.1371/journal.pone.0119445)
Supplement: S1 DataSet — The data file is in Matlab format (.mat) and contains a data structure with, for each subject and for each trial: 1) the gaze coordinates estimated from the eye-tracker recordings as specified in the Methods; 2) the head, the body, and the ball data recorded with the Vicon Motion capture system; 3) the information about the trial characteristics (i.e. the T—Z condition, the catching score, and the launch and impact time events). (ZIP) [file pone.0119445.s001.zip › Supporting_Information/ReadMe.pdf]

data.mat is a Matlab file containing the subject's data structures used in the analysis presented in the manuscript.

The structure is organized according to this organizational chart:

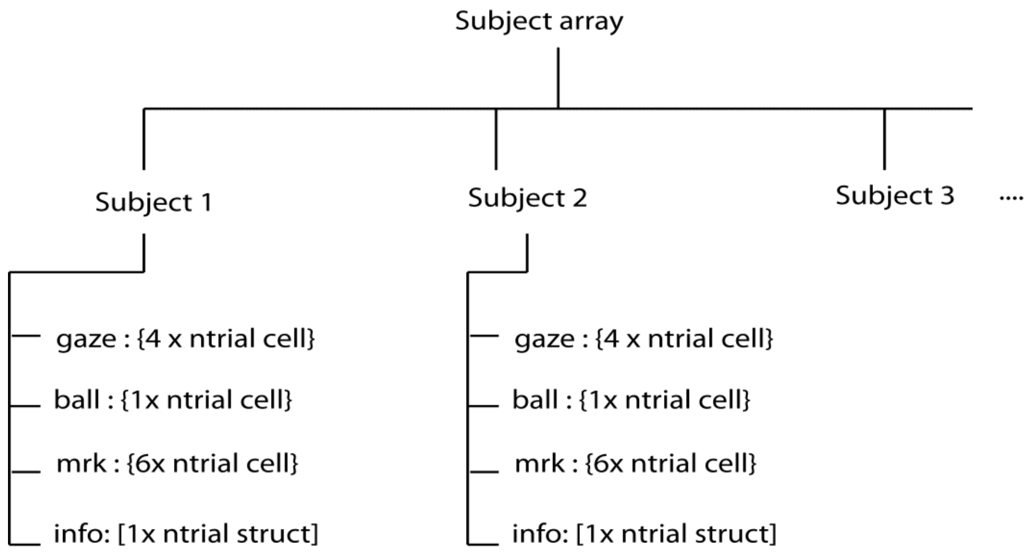

- Gaze field:

4 x ntrial cell. Each cell is relative to a specific trial of the experimental session. Each cell contains 4 structures.

Structure 1: gaze coordinates of the left eye
Structure 2: gaze coordinate of the right eye
Structure 3: ball position converted in the gaze coordinate of the left eye
Structure 4: ball position converted in the gaze coordinate of the right eye
Gaze data are filtered as specified in the methods of the manuscript.
Each structure contains two elements:
x: azimuth and elevation coordinates
t: time samples

- Ball field:

Each cell contains the ball data recorded with the Vicon Motion capture system. Data are relative only to the visible part of the ball during the experiment.
Each structure contains two elements:
x : x, y, z coordinates of the ball
t : time samples;

Data are not filtered

- Mrk

Each cell contains the head and body data recorded with the Vicon Motion capture system. The list of the marker is specified for each ith-trial in the info(itrial).markername field.

Each structure contains two elements:
x : x, y, z coordinates of the marker
t : time samples;

Data are not filtered

- info

Each element of the structure is relative to one trial of the experimental session. Each element contains the following fields:

markernames : list of the marker (ordered as presented in the data{isubj}.mrk field.
itrial: trial number in the experiment
type : 5 element array that specify the trial characteristics
type(1): Time flight condition (1-> 0.55 s; 2-> 0.65s; 3->0.75; 4-> 0.85)
type(2): Ball arrival height condition (1-> low ; 2-> high);
type(3): Score (0 -> caught, 1 -> non-caught);
events: time events of the trial. This element contains two fields:
code : event code (1-> launch time; 2-> impact time)
time : time of the events

Only the selected trials are reported.

Example:

Matlab code to plot the gaze and ball left eye elevation coordinates reported in Figure 2 panel B.

```
tlaunch  = data{9}.info(73).events.time(1);
timpact  = data{9}.info(73).events.time(2);

databall = data{9}.gaze{3,73}
dataeye = data{9}.gaze{1,73};

ind = find(dataeye.t>= tlaunch & dataeye.t<= timpact);
time = dataeye.t(ind) -tlaunch;
plot(time, dataeye.x(2,ind), '.-r');
hold on
plot(time, databall.x(2,ind), '.-b');
```
